# PhylOTU: A High-Throughput Procedure Quantifies Microbial Community Diversity and Resolves Novel Taxa from Metagenomic Data

**DOI:** 10.1371/journal.pcbi.1001061

**Published:** 2011-01-20

**Authors:** Thomas J. Sharpton, Samantha J. Riesenfeld, Steven W. Kembel, Joshua Ladau, James P. O'Dwyer, Jessica L. Green, Jonathan A. Eisen, Katherine S. Pollard

**Affiliations:** 1 The J. David Gladstone Institutes, University of California San Francisco, San Francisco, California, United States of America; 2 Center for Ecology and Evolutionary Biology, University of Oregon, Eugene, Oregon, United States of America; 3 Institute of Integrative and Comparative Biology, University of Leeds, Leeds, United Kingdom; 4 Department of Evolution and Ecology, University of California Davis, Davis, California, United States of America; 5 Institute for Human Genetics & Division of Biostatistics, University of California San Francisco, San Francisco, California, United States of America; Technion-Israel Institute of Technology, Israel

## Abstract

Microbial diversity is typically characterized by clustering ribosomal RNA (SSU-rRNA) sequences into operational taxonomic units (OTUs). Targeted sequencing of environmental SSU-rRNA markers via PCR may fail to detect OTUs due to biases in priming and amplification. Analysis of shotgun sequenced environmental DNA, known as metagenomics, avoids amplification bias but generates fragmentary, non-overlapping sequence reads that cannot be clustered by existing OTU-finding methods. To circumvent these limitations, we developed PhylOTU, a computational workflow that identifies OTUs from metagenomic SSU-rRNA sequence data through the use of phylogenetic principles and probabilistic sequence profiles. Using simulated metagenomic data, we quantified the accuracy with which PhylOTU clusters reads into OTUs. Comparisons of PCR and shotgun sequenced SSU-rRNA markers derived from the global open ocean revealed that while PCR libraries identify more OTUs per sequenced residue, metagenomic libraries recover a greater taxonomic diversity of OTUs. In addition, we discover novel species, genera and families in the metagenomic libraries, including OTUs from phyla missed by analysis of PCR sequences. Taken together, these results suggest that PhylOTU enables characterization of part of the biosphere currently hidden from PCR-based surveys of diversity?

## Author Summary

Microorganisms comprise the majority of the biodiversity on the planet. Because the overwhelming majority of microbes are not readily cultured in the laboratory, researchers often rely on PCR-based investigations of genomic sequence to characterize microbial diversity. These analyses have dramatically expanded our understanding of biodiversity, but due to methodological biases PCR-based approaches may only reveal part of the microbial biosphere. Shotgun sequencing of environmental DNA, known as metagenomics, avoids the biases associated with targeted amplification of genomic sequence and can provide insight into the diversity hidden from traditional investigations. However, the fragmentary, non-overlapping nature of shotgun sequence data makes it intractable to analyze with existing tools. Here, we present PhylOTU, a novel computational method that enables accurate characterization of microbial diversity from metagenomic data. We process over 10 million metagenomic sequences obtained from the global open ocean to identify novel Bacterial taxa and reveal the presence of microorganisms overlooked by investigation of PCR-based sequences from the same samples. These results suggest that to fully characterize microbial biodiversity requires a novel bioinformatics toolbox for analysis of shotgun metagenomic data.

## Introduction

A central goal of ecology and evolution is to understand the forces that shape biodiversity - the variety of life on Earth. It is becoming increasingly clear that global biodiversity is mostly microbial. It is estimated that there are millions of microbial species on the planet, relatively few of which have been isolated in culture [1]–[2]. Despite the recognized importance of microorganisms, we still know little about the magnitude and variability of microbial biodiversity in natural environments relative to what is known about plants and animals. This is a major knowledge gap, given that microbes are critical components of our planet, responsible for key ecosystems services including the production of agriculturally critical small molecules, the degradation of environmental contaminants, and the regulation of human host phenotypes.

Biodiversity science has traditionally focused on comparing species richness across space, time and environments. Out of necessity, microbial diversity studies usually examine the richness (i.e. number) of operational taxonomic units (OTUs), where OTUs are sequence similarity based surrogates for microbial taxa, which can be difficult to define. In addition to richness, OTUs have been used to characterize the abundance, range, and distribution of microbes, thereby improving our understanding of both natural ecosystems and human health [3]–[6]. OTUs are commonly identified by aligning sequences of the small subunit of ribosomal RNA (SSU-rRNA) from one or more samples and identifying groups of related sequences using a hierarchical clustering algorithm. This clustering is based upon a measure of distance between all pairs of sequences, which is typically defined using some variant of the percent sequence identify (PID) (e.g. [3], [7]–[8]). For example, researchers traditionally cluster sequences that are no more than 3% diverged into the same OTU. This designation has been proposed as being roughly equivalent to a species-level classification [9], though evidence suggests that it may result in an underestimate of the true number of species [10].

The SSU-rRNA sequences for OTU identification are traditionally amplified from a sample via polymerase chain reaction (PCR) using universal primers. Each PCR product is then individually sequenced. One of the biggest drawbacks of this targeted sequencing approach is that it leverages PCR, which has been shown to exhibit sequence-based biases at the level of priming and extension [11]–[13]. In addition, the so-called ‘universal’ PCR primers used in such assays will fail to amplify sequences sufficiently diverged from those used to design the primers. The result is that some taxa may be disproportionately amplified or even missed [14]. Metagenomic approaches eliminate this bias by sequencing randomly-sheared fragments (i.e., shotgun sequencing) of environmental DNA, and, despite having their own sources of bias [15], may therefore provide a potentially more accurate characterization of microbial diversity. For example, the analysis of metagenomic data from a relatively simple microbial community revealed the presence of low-abundance acidophilic Archaea overlooked by PCR-based surveys of diversity [16].

Because of the fragmentary nature of shotgun sequencing, metagenomic reads frequently exhibit minimal, if any, sequence overlap. PID-based evaluations using metagenomic data are thus restricted to the subset of reads that mutually overlap and can therefore be aligned to one another (e.g., [17] and [18]). Alternative approaches have been adopted to describe microbial diversity from non-overlapping metagenomic sequences, including the binning of reads into a reference taxon by comparing each read against reference sequence databases (e.g., [17], [19] and [20]) and using *de novo* sequence assemblers to build SSU-rRNA contigs (e.g., [21]). While these approaches have substantially advanced the field of microbial biodiversity, they exhibit significant limitations. The former is limited by the diversity encoded in sequence databases, most of which was obtained via targeted sequencing studies. The latter is restricted to the subset of high-confidence assemblies, which can be difficult to produce in many environments given that contig assembly may result in chimeric SSU-rRNA sequences from complex communities [22]). Despite the rapidly growing metagenomic data in microbial ecology and human microbiome studies, no method currently provides a means of characterizing microbial diversity directly from non-overlapping metagenomic data. There is a great need for new approaches that identify OTUs using metagenomic data.

We present PhylOTU, the first method that enables automated identification of microbial OTUs directly from non-overlapping metagenomic sequence reads. PhylOTU leverages a phylogenetic tree of metagenomic SSU-rRNA reads, constructed using probabilistic sequence profiles built from full-length SSU-rRNA sequences from completed genomes, to identify and characterize phylogenetic distances between SSU-rRNA reads in metagenomic data sets. This phylogenetic distance (PD), rather than PID, is then used to cluster reads into OTUs in a fashion similar to that utilized for targeted sequencing data. Because the enormous volume of sequence in most metagenomic libraries presents substantial challenges in the form of sequence-alignment quality and the rate of computational through-put, we developed and implemented within PhylOTU a series of data quality control filters and efficient data structures. We also developed an error rate metric for the analysis of clustered data and used simulated sequences to quantify the accuracy of PhylOTU. These investigations enabled us to derive corrections for biases in phylogenetic methods, producing a tool with similar accuracy to existing PID-based methods. We used PhylOTU to describe microbial diversity in the global open ocean by processing the 10,133,846 shotgun reads in the Global Ocean Survey sequence library [21]. In addition, we compared the OTUs identified by PCR-generated sequences to those identified by shotgun sequences from the same samples. We find that analysis of shotgun sequences reveals a novel part of the biosphere missed by analysis of PCR-generated sequences. PhylOTU is freely available for download at github (https://github.com/sharpton/PhylOTU) and BioTorrents (http://www.biotorrents.net) [23].

## Results

### A Novel Workflow for Identifying OTUs from Shotgun Data

Traditionally, OTUs are identified from a PCR-generated targeted sequence library by aligning all pairs of sequences, calculating each pair's PID-based distance, and using this distance to group sequences using agglomerative hierarchical clustering. Due to the fragmentary nature of shotgun metagenomic reads, this traditional approach is limited to the subset of overlapping sequences; non-overlapping reads cannot be directly aligned to one another. Even when reads can be aligned (e.g., to full-length reference sequences), one still cannot calculate PID for sequences that do not overlap. To overcome these limitations, we designed PhylOTU, which uses a probabilistic sequence profile to align reads and a phylogenetic tree to infer their similarity.

**Figure 1:**
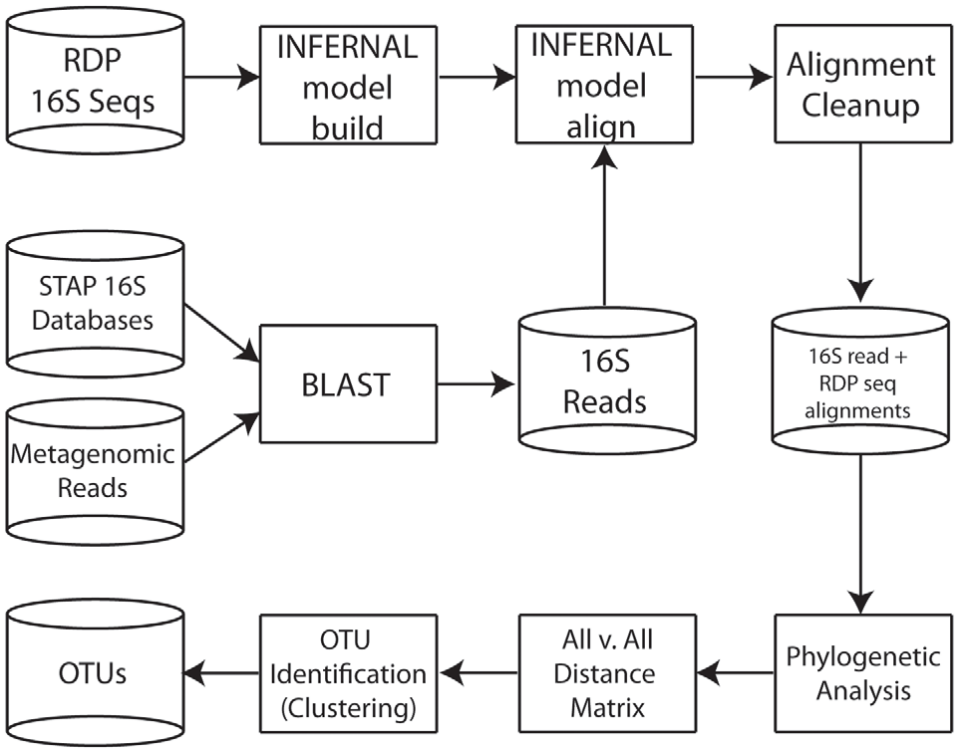
**PhylOTU** Workflow. Computational processes are represented as squares and databases are represented as cylinders in this generalize workflow of PhylOTU. See Results section for details.

The general strategy PhylOTU employs is to leverage full-length reference sequences to construct a probabilistic sequence profile of SSU-rRNA. The profile is used to align metagenomic reads and reference sequences, and this alignment is in turn used to compute the phylogenetic distance between every pair of reads for input into the clustering algorithm. A general workflow schematic of our method is illustrated in Figure 1.

First, probabilistic profiles that encode the evolutionary diversity and secondary structure of the SSU-rRNA sequence from Bacteria and Archaea [24] are constructed via high-quality reference alignments of full-length SSU-rRNA sequence [25]. These profiles are pre-computed for use in different metagenomic analyses. For a given metagenomic data set, SSU-rRNA homologous reads are identified from the shotgun sequencing data via a BLAST search of every metagenomic read against the small but phylogenetically diverse SSU-rRNA STAP databases [26]. This relatively fast search allows one to accurately differentiate SSU-rRNA homologs of Archaea from those of Bacteria, which in turn accelerates and improves downstream alignment and phylogenetic analysis. Multiple sequence alignments of metagenomic reads are created by aligning each SSU-rRNA read to the appropriate Bacterial or Archaeal SSU-rRNA profile, using profile alignment methods [24]. This read alignment is then mapped onto the reference alignment used to build the profile, resulting in a multiple sequence alignment of full-length reference sequences and metagenomic reads. The final step of the alignment process is a quality control filter that 1) ensures that only homologous SSU-rRNA sequences from the appropriate phylogenetic domain are included in the final alignment, and 2) masks highly gapped alignment columns (see Text S1).

We use this high quality alignment of metagenomic reads and references sequences to construct a fully-resolved, phylogenetic tree and hence determine the evolutionary relationships between the reads. Reference sequences are included in this stage of the analysis to guide the phylogenetic assignment of the relatively short metagenomic reads. While the software can be easily extended to incorporate a number of different phylogenetic tools capable of analyzing metagenomic data (e.g., RAxML [27], pplacer [28], etc.), PhylOTU currently employs FastTree as a default method due to its relatively high speed-to-performance ratio and its ability to construct accurate trees in the presence of highly-gapped data [29]. After construction of the phylogeny, lineages representing reference sequences are pruned from the tree. The resulting phylogeny of metagenomic reads is then used to compute a PD distance matrix in which the distance between a pair of reads is defined as the total tree path distance (i.e., branch length) separating the two reads [30]. This tree-based distance matrix is subsequently used to hierarchically cluster metagenomic reads via MOTHUR into OTUs in a fashion similar to traditional PID-based analysis [31]. As with PID clustering, the hierarchical algorithm can be tuned to produce finer or courser clusters, corresponding to different taxonomic levels, by adjusting the clustering threshold and linkage method.

To evaluate the performance of PhylOTU, we employed statistical comparisons of distance matrices and clustering results for a variety of data sets. These investigations aimed 1) to compare PD versus PID clustering, 2) to explore overlap between PhylOTU clusters and recognized taxonomic designations, and 3) to quantify the accuracy of PhylOTU clusters from shotgun reads relative to those obtained from full-length sequences.

### PhylOTU Clusters Recapitulate PID Clusters

We sought to identify how PD-based clustering compares to commonly employed PID-based clustering methods by applying the two methods to the same set of sequences. Both PID-based clustering and PhylOTU may be used to identify OTUs from overlapping sequences. Therefore we applied both methods to a dataset of 508 full-length bacterial SSU-rRNA sequences (reference sequences; see above) obtained from the Ribosomal Database Project (RDP) [25]. Recent work has demonstrated that PID is more accurately calculated from pairwise alignments than multiple sequence alignments [32]–[33], so we used ESPRIT, which implements pairwise alignments, to obtain a PID distance matrix for the reference sequences [32]. We used PhylOTU to compute a PD distance matrix for the same data. Then, we used MOTHUR to hierarchically cluster sequences into OTUs based on both PID and PD. For each of the two distance matrices, we employed a range of clustering thresholds and three different definitions of linkage in the hierarchical clustering algorithm: nearest-neighbor, average, and furthest-neighbor.

To statistically evaluate the similarity of cluster composition between of each pair of clustering results, we used two summary statistics that together capture the frequency with which sequences are co-clustered in both analyses: true conjunction rate (i.e., the proportion of pairs of sequences derived from the same cluster in the first analysis that also are clustered together in the second analysis) and true disjunction rate (i.e., the proportion of pairs of sequences derived from different clusters in the first analysis that also are not clustered together in the second analysis) (see Methods and Figure S1). PhylOTU exhibits high true conjunction and true disjunction rates at commonly employed PID thresholds (e.g., 0.03, 0.06), demonstrating that PD-based clustering accurately recapitulated PID-based clustering at the same threshold (Figure S2).

On the other hand, when applying the same clustering threshold to both distance matrices, PID-based clustering produces a higher richness estimate (i.e., total number of OTUs) than PD-based clustering (Table S1). Comparing the pairwise distance distributions obtained from the PID- and PD- based approaches finds that at relatively short distances (e.g., 0–0.03), PD-based pairwise distances are shorter than the corresponding PID-based distances, while at relatively long distances (e.g., greater than 0.1), PD-based pairwise distances are longer than the corresponding PID-based distances (Figure S3). These findings suggest that differences in richness estimates result from the fact that PD-based clustering tends to merge some clusters that are found to be distinct, but closely related, by PID-based clustering. However, the overall composition of the clusters is very similar: merging of closely related clusters results in a significant reduction in estimated richness, but can produce a relatively small number of conjunction and disjunction errors.

We subsequently investigated whether we could both maintain accuracy of PD-based clustering, while at the same time obtaining richness estimates more similar to PID-based results, which are thought to approximately correspond to the number of distinct microbial taxa in an environmental sample. First, we considered changing the hierarchical clustering algorithm. It has been shown that the choice of nearest-neighbor, average, or furthest-neighbor linkage in hierarchical clustering algorithms results in substantially different estimates of taxonomic richness, with average-linkage clustering performing the best for PID-based approaches [33]. In agreement with these earlier studies, we observed different OTU richness estimates when these three different linkage methods were employed in PhylOTU, with furthest-neighbor clustering producing richness estimates most similar to PID-based clustering for a given threshold (Table S1). But there is a trade-off: employing a different clustering algorithm generally reduces the accuracy with which PhylOTU clusters recapitulate PID-based OTUs, implying that while our estimate for richness may be improved by varying the clustering algorithm, we might be finding the right number of ‘wrong’ OTUs. We reach a similar conclusion if we lower the PD-clustering threshold. We naturally find a greater number of OTUs with a lower threshold, so a threshold that produces a PID-like OTU richness estimate can be identified. However, the accuracy of PD clustering relative to PID clustering becomes systematically lower as the PD threshold deviates from the PID threshold. Given these results, PhylOTU implements average-linkage and a threshold of 0.03 as default settings when clustering full-length SSU-rRNA sequences into OTUs (Table 1).

**Table 1:** Clustering thresholds applied to various OTU identification analyses.

PID	PD Full-length Sequence	PD Shotgun Read
0.03	0.03	0.15
0.06	0.06	0.17

Overall, our results imply that PhylOTU finds OTUs very similar to PID-based methods in terms of cluster composition, but that recapitulating PID-based clusters with high accuracy will generally result in a lower richness estimate. We consider the accurate clustering of sequences to be more critical than matching OTU richness, given that an equal number of clusters may be optimized between two methods while the accuracy of cluster member composition is simultaneously low. Therefore, we recommend using the default PhylOTU settings, which optimize similarity to PID-based clusters, with the caveat that lower OTU richness estimates may be produced.

### PhylOTU Produces Taxonomically Meaningful Clusters

Next, we looked at how well PhylOTU clusters full-length sequences relative to taxonomy-guided clusters. We obtained the GenBank taxonomy information for each of the 508 full-length reference sequences and clustered them into taxonomic groups at the species level. We find that PhylOTU clusters sequences into their proper taxonomic group with high true conjunction (96.5%) and true disjunction (99.4%) rates at a clustering threshold of 0.03 (Table S2). However, similar to the results observed in the comparison with PID-based OTUs, PhylOTU tends to underestimate richness relative to GenBank taxonomy. To provide a reference for understanding these results, we conducted a similar comparison of PID-based OTUs and taxonomic groups. PID and PD clustering recapitulate taxonomic groups with similar accuracy at a clustering threshold of 0.03. But, PID clustering produces a slightly closer approximation of richness relative to the taxonomy clusters, consistent with our direct comparison between PhylOTU and PID-based OTUs (Table S2). The similarity between taxonomy and PID-based OTUs is not surprising given the fact many bacterial taxa were defined via PID-based clustering of SSU-rRNA sequences (see Discussion).

### PhylOTU Accurately Clusters Shotgun Reads

To investigate the performance of PhylOTU on metagenomic reads versus full-length sequences, we generated 25 distinct simulation data sets using metaPASSAGE (Riesenfeld et al., unpublished communication), a recently developed, highly parameterized simulation pipeline which expands the function of the MetaSim program [34]. For each simulation, 50 of the 508 reference SSU-rRNA sequences were drawn at random to represent taxa detectable in the sample. These 50 sequences are termed “source sequences” because they are used to generate the simulated metagenomic data. Since most taxa in nature do not have full-length SSU-rRNA sequences in current databases, we used only the remaining 458 non-sampled sequences as the reference sequences for each simulation. We designated the 50 source sequences as full-length PCR products to simulate a targeted sequencing study for each simulated sample. To simulate metagenomic sequencing of the same sample, we generated *in silico* shotgun reads from the 50 source sequences with a read length distribution chosen to be similar to a 454-sequence library (see Methods). We simulated exactly one read per source and did not simulate sampling or PCR bias to enable direct comparison of full-length and shotgun PhylOTU results. For each *in silico* sample, we separately applied PhylOTU to the 50 metagenomic reads and the 50 full-length sequences. We used two metrics to quantify the performance of PhylOTU on metagenomic reads: 1) similarity between the read and full-length sequence distance matrices, and 2) accuracy at which the algorithm clusters reads into OTUs relative to clusters built from full-length sequences.

Comparing the PD matrices from metagenomic and full-length data sets, we observe a strong correlation between the pairwise distances computed on reads and full-length sequences. For each of the 25 simulated samples, the read and corresponding full-length-sequence distance matrices show a positive and significant correlation (Mantel test, p<0.05; Figure S4). Having established that pairwise PD measurements are on average similar between metagenomic reads and full-length sequences, we next investigated whether specific properties of individual metagenomic reads systematically generate errors in metagenomic PD estimates compared to full-length PD measurements. We hypothesized that PD error might be higher in shorter reads, which contribute less phylogenetic information than longer sequences, and in reads from hyper-variable regions in the SSU-rRNA locus, which will have higher than expected substitution rates. To explore these hypotheses, we calculated, for each read, a measure of the relative contribution by that read to the total PD error (see Methods). This measure is designed to detect whether certain reads are placed on particularly poorly estimated parts of the phylogeny. We compared this relative error to read length, location within the SSU-rRNA locus (mapped through a read's midpoint position in the multiple sequence alignment), and the amount of alignment overlap the read shares with other reads. We detected no significant correlation between relative PD error and rate variation or alignment depth. We did find a slightly negative, but significant, correlation between relative PD error and read length, suggesting that short reads may contribute more error than long reads (Spearman's rho  = −0.088, p = 0.0028). This signal disappeared when reads less than 100 base pairs (bp) were removed from the analysis. As a result, we incorporate a 100 bp read length cutoff in our method. Further analyses are required to comprehensively study the effects of read length and other attributes on PD estimates.

**Figure 2:**
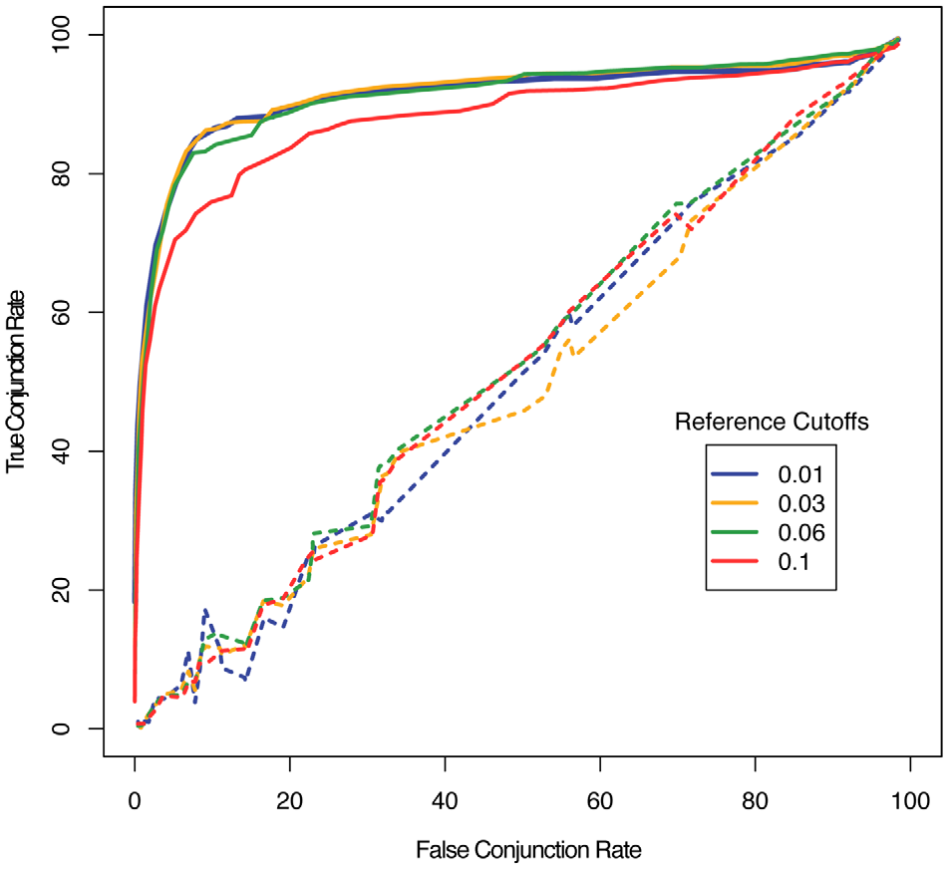
Relationship between false clustering rate and true clustering rate. Each read data set was clustered into OTUs at various thresholds and compared to the corresponding full-length data set, which was clustered at several fixed PD thresholds (shown here are full-length sequence cutoffs of 0.01, 0.03, 0.05 and 0.1). For each full-length sequence threshold, the true conjunction and false conjunction rates of the read OTUs were calculated as a function of the read threshold. Solid lines represent the median value of the true and false conjunction rates across simulations. Dashed lines represent the median value of the true and false conjunction rates derived from comparisons of randomly permuted clusters relative to the source sequence clusters.

Next, we compared the OTUs produced from metagenomic and full-length sequences, using PhylOTU with identical clustering settings. As illustrated in Figure 2, this analysis reveals that even at low false conjunction rates (meaning that few reads whose corresponding full-length sequences are in separate OTUs are clustered together), PhylOTU tends to correctly put reads from the same OTU in the full-length analysis into the same cluster. This indicates that PhylOTU accurately discriminates between sequence-pair conjunctions: false conjunctions do not need to be tolerated at a high rate to identify true conjunctions. Additionally, PhylOTU clusters reads substantially better than randomly permuting reads into OTU clusters.

We then determined whether the performance of PhylOTU on metagenomic data could be improved by tuning the parameters of the clustering algorithm. Taking the OTUs from full-length sequences at a given clustering threshold as a gold standard, we explored how the true conjunction rate and true disjunction rate vary as functions of the threshold used to cluster the reads. There exists a tradeoff between the true conjunction and true disjunction rates as the threshold changes: at small threshold values, PhylOTU accurately separates reads into distinct OTUs, while at high threshold values, the algorithm accurately clusters sequences into the same OTU (see Figure S5). Maximizing the true disjunction rate subject to a minimum true conjunction rate of 80%, we observe that increasing the read threshold relative to the full-length sequence threshold greatly improves the agreement between the two sets of OTUs. Interestingly, we find a nearly linear relationship between the most accurate read clustering threshold and the full-length sequence threshold (Figure S6). This relationship and the accuracy of PhylOTU remains consistent up to relatively large full-length sequence clustering thresholds (e.g., 0.29, Figure S7). The linear relationship between read and full-length sequence thresholds enabled us to identify adjusted thresholds for metagenomic reads that accurately recapitulate OTUs from full-length sequences (Table S3). PhylOTU obtains 80% accuracy (true conjunction rate  = 80%, true disjunction rate  = 99.58%) at a read threshold of 0.09, and 90% accuracy (true conjunction rate  = 90%, true disjunction rate  = 98.73%) at a threshold of 0.18. Thus, simulations enabled us to select tuning parameters of the hierarchical clustering algorithm in PhylOTU so that the OTUs generated from shotgun read data closely resemble those that would be identified if full-length PCR products were available for each SSU-rRNA sequence in the read library.

Given this insight into the accuracy with which PhylOTU clusters metagenomic reads under relatively simple simulation parameters, we evaluated how PhylOTU performs using more rigorous parameters that are reflective of situations encountered during real studies. First, in some environmental samples, the average read may be quite diverged from its closest reference sequence. Second, in many studies the number of reads will be greater than the number of reference sequences. To investigate these two issues, we first used our simulated sequences to evaluate the relationship between the mean phylogenetic distance from each read to its nearest reference sequence (e.g., read-to-reference distance) and the true conjunction rate. We found no significant correlation (Spearman's test). Next, we conducted additional simulations based on sampling reads from full-length Bacterial SSU-rRNA sequences in the SILVA database [35]. This investigation allowed us to generate data sets with more reads than reference sequences and where read-to-reference distances exceeded those in our primary simulations. The latter property is important because of known phylogenetic sampling biases, especially for sequenced genomes [36]. For each of 15 independent simulations, we randomly sampled 1,000 SSU-rRNA sequences from the SILVA database, reflecting the approximate number of SSU-rRNA reads expected when performing one run of next-generation sequencing on a shotgun library. These 1,000 source sequences were then used to simulate metagenomic reads as described above. Reference sequences were pruned from both the source and simulation phylogenies and full-length source sequences and simulated reads were then clustered into OTUs. In these simulations, the average distance between each read and its nearest source is an order of magnitude greater than that observed in our previous simulation analysis (0.182 versus 0.010 mean read-to-reference distance), which is expected given that the SILVA database is highly populated and comprised of phylogenetically diverse sequence data. Evaluating the accuracy of PhylOTU under these conditions reveals high true disjunction rates, similar to those observed in the RDP reference library based simulations. True conjunction rates are somewhat lower, but still meet our accuracy standards. For example, at a read threshold of 0.15, PhylOTU clusters metagenomic reads with an 80% true conjunction rate and a 98.8% true disjunction rate (Figure S8, Table S4), when compared to full-length sequences clustered at a threshold of 0.03 (corresponding to an 86.8% true conjunction rate and a 98.8% true disjunction rate under RDP reference library based simulation parameters). This suggests that read library size and phylogenetic novelty do have a small impact on the accuracy of PhylOTU, but that they can generally be compensated for by appropriately tuning the clustering cutoff.

### PhylOTU Reveals Novel Taxa from Global Ocean Survey Reads

To demonstrate the utility of PID-based clustering of metagenomic data, we analyzed the pooled Global Ocean Survey (GOS) metagenomic read library [21] with PhylOTU. This data set represents the most extensive publicly available metagenomic sequence library generated to date, with the exception of the Illumina library generated by Qin et. al, which contains reads that are too short to process via PhylOTU [37]. Additionally, many of the GOS sampling sites were also explored with deep, targeted sequencing of the SSU-rRNA locus enabling comparisons of shotgun and PCR libraries. Despite the use of Sanger sequencing, the mean SSU-rRNA metagenomic read length is roughly similar to that used in our simulation analysis (518 bp). Thus, the GOS read library represents the best opportunity to explore PhylOTU's ability to discover novel taxa from metagenomic data. Of the 10,133,846 Sanger sequenced reads in the library, PhylOTU identifies 14,320 Bacterial SSU-rRNA homologs, of which 12,020 passed the method's filters and could be used for OTU discovery. Previous work using the same library was constrained to analysis of 4,125 high-confidence SSU-rRNA assemblies [21], the difference resulting from the fact that many of the SSU-rRNA reads identified by PhylOTU were either assembled in this prior analysis or excluded from this early work given assembly constraints. PhylOTU clusters the 12,020 SSU-rRNA reads into 833 OTUs at a PD threshold of 0.15, which, according to our SILVA-based simulation analysis, corresponds to a full-length threshold of 0.03. Applying a cutoff of 0.09, which was identified as the appropriate corresponding cutoff from the RDP reference library based simulations, identifies 1,078 OTUs. We also identify 192 Archaeal SSU-rRNA sequences, 79 of which pass the quality control filters and cluster into 7 OTUs when using the 0.15 threshold and 10 OTUs when using a threshold of 0.09. This compares to the 811 total OTUs identified by Rusch et. al. via analysis of assembled SSU-rRNA reads at the 97% identity level. We have made our designation of OTUs derived from GOS metagenomic reads and PCR sequences available at BioTorrents [23]. This comparison reveals the ability of assembly-free methods such as PhylOTU to identify novel taxa missed by approaches that rely upon assembled contigs.

**Figure 3:**
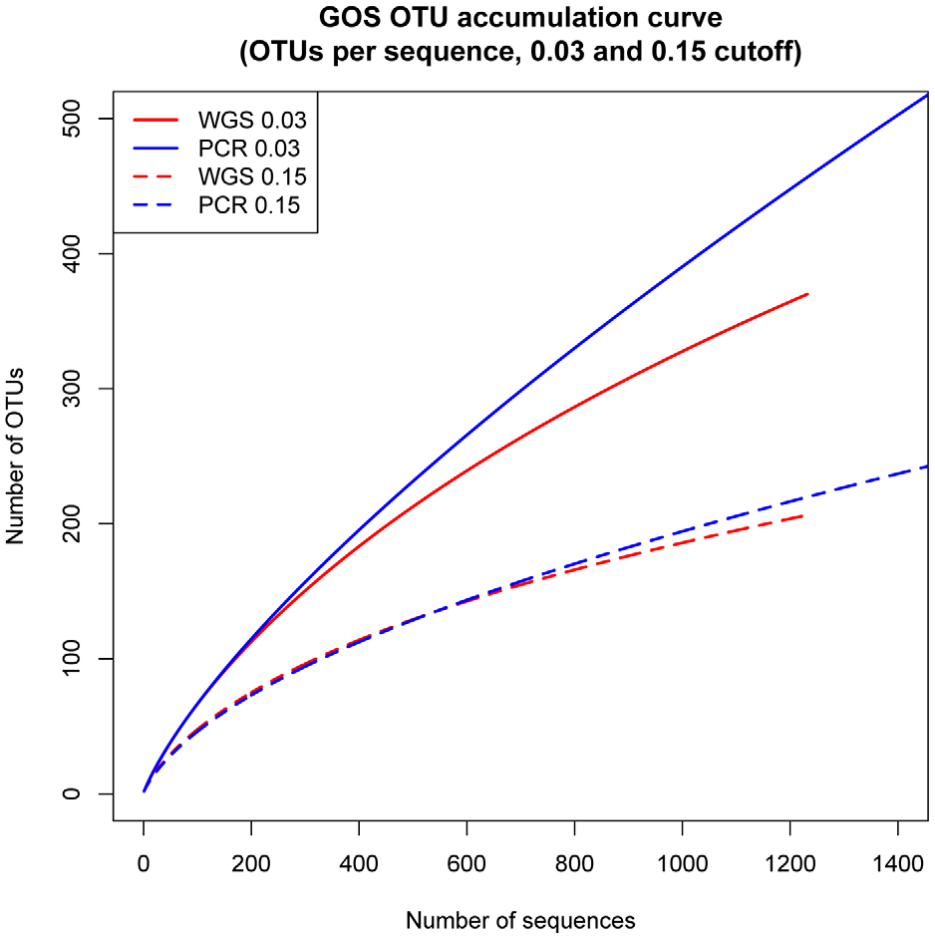
Rarefaction analysis of OTUs identified from PCR and metagenomic sequencing at two different sequence similarity cutoffs (solid = 0.03, dashed = 0.15). Rarefaction curves are shown for OTUs from PCR (blue) and metagenomic (red) sequencing libraries. Two different sequence similarity cutoffs are used (solid = 0.03, dashed = 0.15). Curves represent the average number of OTUs per sequence from 100 random draws of subsets of sequences from each data set.

The GOS project also generated 6,413 full-length SSU-rRNA sequences via targeted sequencing of PCR products from six of the 73 geographical sites surveyed [38]. We evaluated the ability of PhylOTU to discover novel taxa in shotgun data by comparing the OTUs identified from metagenomic reads to those identified from full-length PCR data from these six sites. We applied PhylOTU to both data sets and corrected for the difference in sequence types by adjusting the read threshold relative to the full-length sequence threshold according to our simulation analysis. Specifically, we used a read threshold of 0.15 and a full-length sequence threshold of 0.03 to evaluate diversity at approximately the species level. We compared the number of OTUs identified per sequence across methods by conducting a rarefaction analysis (Figure 3) [39]. For each method and for subsets of the full data set from one to the observed number of sequences, we drew 100 random subsets of sequences from each data set and calculated the average number of OTUs identified by each method for that number of sequences. This allowed a comparison of the effect of read threshold and sequencing method on the total number of OTUs and rate of OTU accumulation. While there are more PCR SSU-rRNA sequences (N = 6,413) and OTUs (N = 1,563) than metagenomic SSU-rRNA reads (N = 1,233) or OTUs (N = 242), when normalized for the number of sequences in each library, the number of OTUs identified per sequence are similar for the two libraries (0.24 for PCR sequences, 0.20 for shotgun sequences). After normalizing by the average sequence length for each library, however, the shotgun sequence data generates three times as many OTUs per sequenced SSU-rRNA base relative to PCR-generated sequences (4.63×10^−4^ and 1.66×10^−4^ OTUs per sequenced base, respectively).

**Figure 4:**
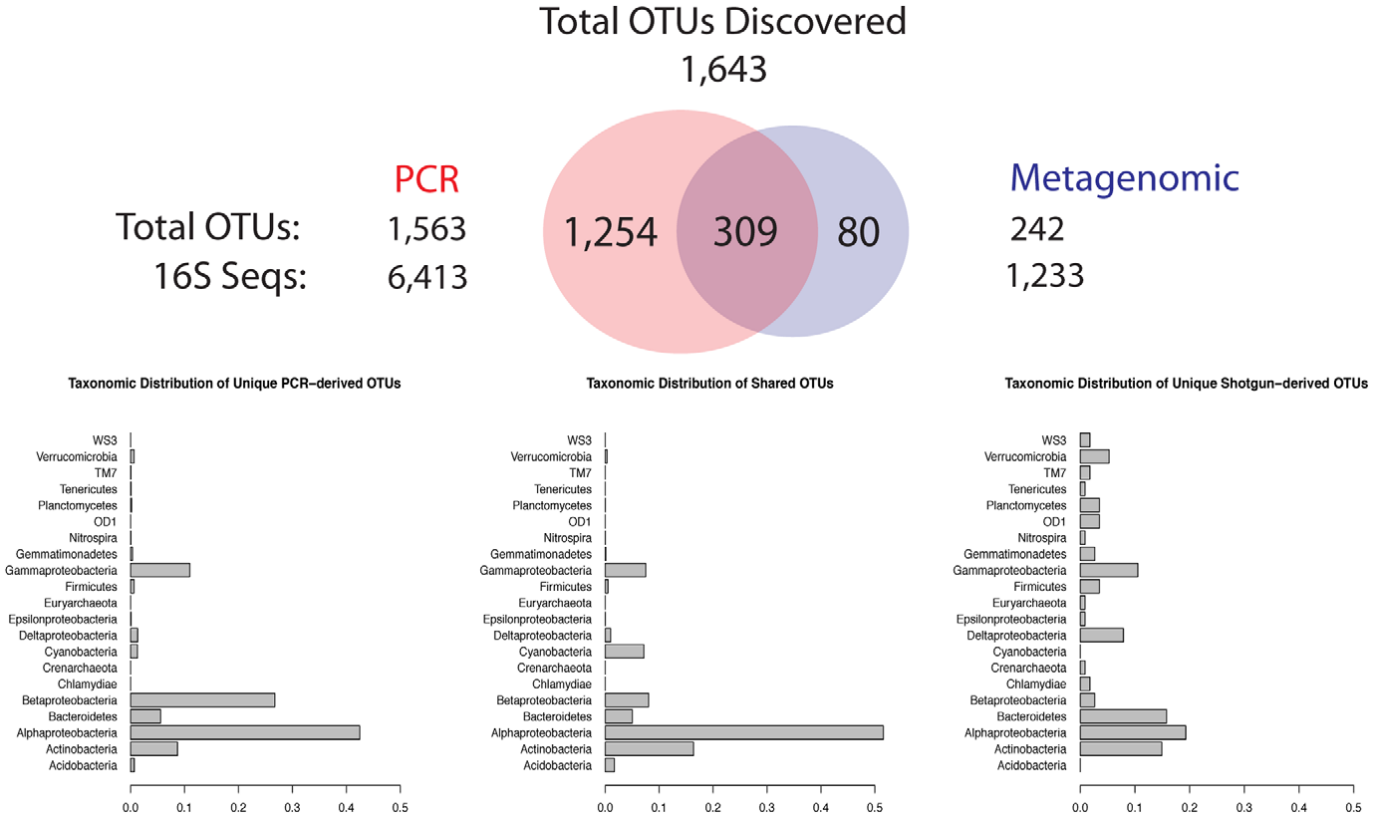
Overlap between GOS OTUs revealed by PCR sequencing and metagenomic sequencing. PhylOTU was used to identify OTUs from a data set comprised of both PCR and shotgun SSU-rRNA sequences obtained from six Global Ocean Survey samples. The 1,254 OTUs that contained only PCR sequences at a clustering threshold of 0.03 were designated as OTUs unique to PCR, while those 80 OTUs that contained only metagenomic sequences at the corresponding clustering threshold of 0.15 (see Results) were designated as OTUs unique to metagenomic sequencing. The 309 OTUs identified by both PCR and shotgun sequencing was determined by using a clustering threshold of 0.03 (162 shared OTUs are identified when a threshold of 0.15 is used). The total number of OTUs and the total number of SSU-rRNA sequences are shown on the left and right of the Venn-Diagram for the PCR (threshold = 0.03) and metagenomic data (threshold = 0.15), respectively. The taxonomic distribution of each set of OTUs is shown beneath the Venn-Diagram. Here, every sequence from each OTU was taxonomically classified into major clades of Bacteria (approximately phylum level designations) using the Ribosomal Database Project classification software. The relative abundance of each taxonomic group is plotted along the x-axis (specific values can be found in Table S5). Clades exhibiting less than 1% relative abundance across all sets of OTUs are not shown.

Evaluating the intersection of OTUs identified by the two libraries when they were pooled together and processed by PhylOTU reveals a shared set of OTUs as well as unique OTUs missed by each method (Figure 4). Because this pooled data set contains both full-length sequences and shotgun reads, we evaluated the distribution of sequences across OTUs for a range of thresholds (Figure S9) and made comparisons between OTUs obtained at thresholds appropriate for full-length sequence (0.03) and shotgun reads (0.15). Specifically, at the 0.15 threshold, the metagenomic library contains 80 OTUs that are not revealed through analysis of the PCR library, while the PCR library contains 1,254 unique OTUs at the 0.03 threshold. Normalizing the number of unique OTUs by the number of sequences per library finds that the PCR-based sequences encode more unique OTUs per sequence (0.19) than shotgun sequences (0.06). However, comparing the change in the number of OTUs uniquely identified by shotgun sequence data to the change in the number of OTUs uniquely identified by PCR sequence data across thresholds suggests that shotgun sequences reveal unique OTUs that are highly diverged from those identified using PCR-based sequences (Figure S9). Despite the amount of sequencing conducted, the steep slopes of the rarefaction curves indicate that sampling has not been saturated at these geographical sites. Thus, deeper sequencing through either method is warranted and may either increase or reduce the number of unique OTUs.

We compared the sequences from the novel OTUs identified from metagenomic reads to the Greengenes SSU-rRNA sequence database to determine if any other PCR-based study revealed the existence of these taxa [40]. Using traditional percent identity cutoffs and the Greengenes database as a reference of nearest neighbor percent identity (e.g., DNAML distance), we find that many of the metagenomic read OTUs represent novel species, genera and families. We further characterized the taxonomic distribution of these novel OTUs via taxonomic classification through comparison of the sequences to the RDP database. OTUs unique to the metagenomic reads are predominantly members of the Alpha- (19%) and Gamma-proteobacteria (11%), Actinobacteria (15%), and Bacteroidetes (12%). We also find that the Bacteroidetes, Verrucomicrobia, Firmicutes, and Delta-proteobacteria are enriched in the OTUs unique to shotgun sequences relative to OTUs unique to PCR data or shared between metagenomic and PCR data (Table S5). In addition, several clades, including TM7, Planctomycetes, OD1, and WS3 were only identified via analysis of metagenomic sequence. Reasoning that the universal PCR primers traditionally employed in most targeted sequencing studies (i.e., 8F, 27F, 1525R, 1429R [41]–[43]), may inefficiently amplify or fail to amplify the SSU-rRNA sequences uniquely identified via shotgun sequences, we searched SSU-rRNA reads that overlap the universal priming sites for the presence of sequence complementary to universal SSU-rRNA primers. Of the shotgun reads that overlap a universal priming site (N = 6), we find two that share a unique point mutation relative to the remaining overlapping reads and the 8F and 27F primer sequences (Figure S10). Prior work demonstrated that differences between the primer and template sequences can result in PCR amplification bias [43]. Our findings support the use of universal-primer-sequence variants that include degenerate positions, such as those described in [43], to improve the resolution of lineages harboring this variant through PCR-based investigations. For the remaining reads that do contain a universal priming site, we do not know if the sequence they were generated from contains the anti-sense priming site because these reads do not span the length of the SSU-rRNA locus. Alternatively, these reads may have been obtained from discontinuous rRNA, such as the rRNA sequence found in the mitochondria of *Chlamydomonas* [44]. Should the priming sites be located in relatively disparate parts of the genome, discontinuous rRNA may fail to amplify even if the universal primer sites are highly conserved.

## Discussion

We have developed a novel method that enables comparison of non-overlapping metagenomic SSU-rRNA reads and their assignment into OTUs. This is the first automated procedure that identifies OTUs directly from non-overlapping metagenomic reads, which facilitates the identification of taxa potentially overlooked by targeted sequencing studies and leverages the vast quantities of shotgun sequencing data currently being produced by environmental and microbiome studies. The key innovation allowing us to compare non-overlapping reads is our use of phylogenetic distance (PD) to cluster reads into OTUs in place of PID. Building a phylogenetic tree requires that at least some of the sequences within the input alignment overlap. Thus, we incorporate high-quality, full-length reference sequences into the SSU-rRNA sequence alignment to guide the phylogenetic placement of metagenomic reads. The accuracy of this approach is constrained, at least in part, by the phylogenetic diversity of the reference sequences and the means by which the phylogenetic algorithm processes missing data. For example, it is challenging to assess distances between non-overlapping shotgun reads derived from a similar place in the phylogeny, even via comparison to full-length reference sequences. We determined the robustness of our method by evaluating the OTU assignment accuracy of simulated metagenomic reads relative to their full-length sources, finding that the relative PD between a pair of reads is on average highly consistent with the relative PD between full-length sources. This result indicates that metagenomic reads can be assigned to OTUs with high accuracy by simply scaling the clustering threshold.

We also tested whether clustering based on PD could accurately recapitulate clustering based on PID for full-length reads where both methods may be applied. Processing 508 full-length reference sequences via both algorithms reveals that PD accurately assigns sequences into OTUs when compared to the PID OTUs. However, this analysis also reveals that PD results in lower richness estimates relative to PID. This phenomenon appears to be due to a difference in the relative distances between sequences. Specifically, the phylogenetic approach appears to shorten the estimated distance between closely related sequences, relative to the PID approach. This is likely due to the fact that the PD approach employs a weighted substitution model when calculating distances, while the PID approach treats all substitutions with equal weight. Thus, while the hierarchical structure of the clusters is generally consistent between the two methods, as revealed by the cluster composition accuracy analysis, sister OTUs in the PID analysis tend to be merged together via the PD approach. For this reason, it may be necessary to take into account this systematic difference in order to compare the diversity results from a PD-based study with a PID-based study.

A similar pattern is observed when the PD-based and PID-based OTUs are compared to OTUs constructed from GenBank taxonomy terms. Specifically, both methods accurately cluster the 508 full-length reference sequences at the species and genus level. Both methods also tend to underestimate the richness, though PID produces an estimate more in line with the taxonomy-guided clusters. Though this analysis serves as a useful benchmark, a more thorough investigation of richness estimation may be warranted in future work for several reasons. First, GenBank taxonomy terms do not necessarily recapitulate the true taxonomic signal or correspond to monophyletic clades. Second, there are known errors in taxonomic assignment and annotation of GenBank sequences [45]–[46]. In addition, many of the taxonomy terms found in GenBank were identified by using the PID approach to classify sequence data. As a result, the reference used in this comparison is necessarily biased towards the PID approach. Regardless, this analysis exemplifies the fidelity with which PhylOTU clusters sequences relative to a commonly adopted interpretation of taxonomy.

Having demonstrated the accuracy with which sequences, both full-length and shotgun, are clustered into OTUs using PD, we applied PhylOTU to the Global Ocean Survey (GOS) metagenomic library. Previous characterizations of SSU-rRNA diversity found in the GOS library were limited to full-length sequences amplified via PCR and full-length contigs produced from high-confidence read assemblies [21]. To demonstrate the ability to discover novel taxa directly from metagenomic data, we compared the PD-based OTUs from full-length PCR sequence to those identified from metagenomic reads. Several conclusions can be drawn. First, targeted sequencing produces more SSU-rRNA sequence per sequenced base (since much of the metagenomic library targets other genes), but fewer OTUs per sequenced SSU-rRNA base compared to metagenomic sequencing. Second, metagenomic sequences analyzed via PD reveal taxa missed by the targeted sequencing study. In particular, PhylOTU clusters metagenomic reads into OTUs belonging to several Bacterial Phyla overlooked by the PCR-generated sequences. We were not able to detect the presence of completely conserved universal PCR priming sites for some of these sequences, which supports the theory that some faction of the microbial biosphere may be hidden from the view of PCR-based investigation. Deeper sequencing of either library could erode the signal of library-specific OTUs. Nonetheless, the distinct taxonomic composition of the metagenomic-only OTUs compared to the shared and PCR-only OTUs (Figure 4, Figure S9, and Table S5) supports the hypothesis that the shotgun libraries would continue to contain unique diversity even after deeper sequencing of both libraries. Thus, we conclude that there are real differences in the identified diversity and composition of these communities depending on the sequencing method employed.

Metagenomic sequencing is an increasingly common means of investigating microbial communities. We expect methods, such as PhylOTU, which enable analysis of unassembled, non-overlapping reads to play an important role in the progress of this field. Future developments will include robust characterization of sources of phylogenetic error to improve methodological accuracy, optimization of PD-based richness estimations in conjunction with optimized cluster composition, and the inclusion of more sophisticated phylogenetic algorithms. Additionally, because the output of PhylOTU includes estimates of abundances for the resulting OTUs, future developments will explore the possibility of using PhylOTU to conduct weighted analyses of community structure by incorporating these abundance estimates. We also anticipate that our phylogenetically-based framework can be expanded beyond its current application to improve OTU identification in several ways, including the incorporation of phylogenetic structure and the utilization of multiple loci when designating of OTUs. When coupled with PCR-based sequencing investigations, this type of bioinformatic analysis of metagenomic data should result in a more comprehensive view of microbial biodiversity.

## Methods

### General Methodology

#### Identification of SSU-rRNA from metagenomic data

Metagenomic reads were identified as SSU-rRNA homologs and classified into their appropriate phylogenetic domain in the following manner. First, each read was compared to the full-length SSU-rRNA sequences found in the Bacteria and Archaea STAP SSU-rRNA databases [26] via BLASTn. Reads exhibiting a local alignment with any STAP sequence with an e-value less than 10^-6^ were designated as containing SSU-rRNA homologous sequence. These reads were assigned to either the Archaea or Bacteria based on the phylogenetic domain assignment of the read's top scoring BLAST hit. Finally, each read was trimmed to SSU-rRNA sequence based on the local alignment boundary of its top hit.

#### Multiple sequence alignment of SSU-rRNA sequences

Evolutionary profiles based on stochastic context-free grammars of the SSU-rRNA gene were produced for both the Bacteria and Archaea using INFERNAL [24]. The Ribosomal Database Project's (RDP) hand-curated reference alignments [25] were used for profile training. Trimmed SSU-rRNA reads were aligned to the appropriate profile via cmalign using the rf, dna, hbanded and sub options. These alignments were simultaneously stitched into the corresponding reference alignment through the withali option in cmalign, producing a multiple sequence alignment of SSU-rRNA reads and full length reference SSU-rRNA sequence. Alignments were subject to quality control prior to downstream analysis by 1) masking columns that encoded over 75% gaps, 2) removing any sequence where over 15% of its characters corresponded to an insert state relative to the profile, 3) removing any sequence with over 50 internal (sequence bounded) gap characters, and 4) removing any sequence whose cmalign score per residue was less than 1 (Text S1).

#### Identification of OTUs

Quality controlled alignments were subjected to phylogenetic analysis via FastTree using the dna and pseudocounts options [29]. Importantly, FastTree goes beyond assigning reads to lineages (i.e., “phylotyping”) and generates a fully-resolved tree from which branch lengths can be extracted. Phylogenetic lineages representing full-length reference sequences were pruned from the trees and the total path length between all remaining pairs of lineages was used to create all-versus-all PD distance matrices. PD distance matrices were then queried by MOTHUR [31] to hierarchically cluster SSU-rRNA reads into OTUs using average linkage. Other linkage methods (nearest- and furthest-neighbor) were also explored.

### Simulation Methodology

#### Generation of simulation data

The 508 full-length RDP Bacterial reference SSU-rRNA sequences served as the pool for simulated data. Simulated reference data sets were generated by randomly sampling without replacement 423 of the RDP sequences. This was done five distinct times to create five different simulated reference data sets, or batches. For each batch, the remaining 85 full-length sequences were appropriated as source sequences. Simulated reads were generated from each batch of source sequences five times, resulting in five simulation sets for each of the five batches (a total of 25 simulation data sets). To account for reads that run off the ends of the SSU-rRNA gene, we also created simulations, with the same settings, in which source sequences were concatenated to a 500-bp poly-N sequence pad at the 5′ and 3′ ends, before generating reads. We additionally created simulations from 1000 full-length Bacterial SSU-rRNA sequences drawn at random from the SILVA database [35] to explore the robustness of PhylOTU's accuracy to increased phylogenetic diversity and read abundance.

For a single simulation set, source sequences were used to simulate an average of 5 reads per source via MetaPASSAGE (Riesenfeld et al., unpublished communication), which utilizes MetaSim [34] (see Text S2 for specific settings). Each simulated data set was processed by PhylOTU through the alignment quality-control stage. Simulated reads that passed the quality-control filter were subjected to additional random subsampling to obtain data sets containing one simulated read per source sequence. For the simulations based on the reference sequence database, the remaining 35 source sequences, i.e., those sources not represented in the final set of simulated reads, were added back to the reference data set for that simulation.

#### Analysis of methodological accuracy

Each set of simulated reads and their corresponding full-length source sequences were independently partitioned into OTUs using PhylOTU. We assessed the extent to which the OTUs based on simulated reads recapitulated the OTUs based on full-length sequences as follows. We noted that two types of errors (relative to the full-length clustering) occur when partitioning the simulated reads into OTUs: they can be incorrectly grouped together in the same OTU (false conjunction), or they can be incorrectly separated into different OTUs (false disjunction). To specify the nature of these errors more precisely, let 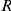
be an arbitrary set of simulated reads, and 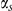 and 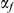 two clustering thresholds. We constructed two partitions of 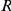 into OTUs: First, we partitioned 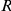 using PhylOTU on input 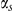. Second, we assembled a set of full-length sequences corresponding to 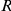, and partitioned this set using PhylOTU with input 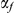; the second partition consisted of the partition of simulated reads corresponding to this partition. Thus, the second partition incorporated information contained in the corresponding full-length sequences, and could be treated as the “correct” partition of 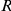 into OTUs. In general, our method for quantifying error rates is applicable whenever two partitions can be constructed, one of which is known to be correct. Let 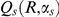 and 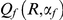 be the equivalence relations (specified as subsets of 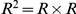) corresponding to these partitions, respectively. Then the rate of false conjunctions for *R* is given by
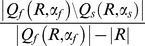
(1)


and the rate of false disjunctions is given by
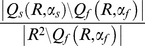
(2)


Note that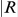 is subtracted from the denominator in Expression (1) because of the reflexivity of equivalence relations.

*R* is a random quantity (generated by a stochastic process), so the quotients in Expressions (1) and (2) are random variables. The error rates of PhylOTU at the threshold values 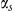 and 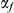 are the expectation values of these random variables: the first expectation value, which we refer to as 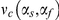, gives the average false conjunction rate at thresholds 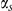 and 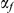, while the second, 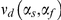, gives the average false disjunction rate at thresholds 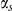 and 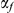 . The error rates 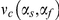 and 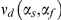 can be calculated for any algorithm used to construct OTUs from threshold values. Moreover, the accuracy of PhylOTU is given by one minus these error rates. Figure S1 provides an example of how these rates are calculated.

We checked whether the PhylOTU algorithm outperformed the following clustering random algorithm:

1. Given a threshold value, partition reads into OTUs using the PhylOTU algorithm.
2. Randomly permute the OTU assignments of the read.

We expected that this algorithm would have much higher error rates than the PhylOTU algorithm; if not, a substantial deficiency in the PhylOTU algorithm would be indicated.

#### Calculation of per-read PD error

The weighted path difference metric [47], an established means of comparing phylogenetic distance matrices, was used to derive the relative PD error contributed by each simulated read. The weighted path difference 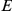 between the source distance matrix 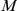
and a simulated read distance matrix 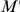, is defined as a scaled Frobenius norm of 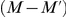, i.e., 
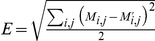



where 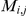 is the PD between full-length source sequences 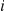 and 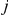, and 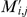 is the PD between the corresponding simulated reads 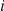 and 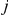. (Note that 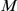 and 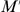 are symmetric matrices with zeros as diagonal entries.) We define the *relative PD error* 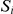 to be the relative contribution to the square of the weighted path difference by read 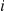, that is:
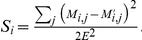



The normalization implies that 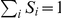, i.e., the relative PD error for all reads sums to 1.

#### Implementation

PhylOTU is written in Perl and requires, in addition to the aforementioned software packages, various R packages plus Perl and BioPerl modules. A full list of requirements can be found on the software download website at github (https://github.com/sharpton/PhylOTU).

## Supporting Information

Figure S1 Example showing how false conjunction and disjunction rates are calculated. In the example, two samples of reads are possible, one consisting of reads A, B, C, D, and E, and another consisting of reads C, D, F, G, and H. In reality, the set of possible samples and reads would be much larger, but for simplicity, we have chosen this smaller set. For the purposes of this example, we suppose that the probabilities of observing samples I and II are 0.3 and 0.7, respectively. At the top of each panel in grey, the partitions into OTUs based on the full length sequences are shown, while the partitions based on the simulated reads are shown in blue. The conjoined and disjoined columns give the pairs of reads placed in the same OTU and different OTUs, respectively, for the partition based on the full-length sequences. The pairs highlighted in blue are correctly conjoined or disjoined in the sample partitions. The rates of false conjunction for Samples I and II are 3/4 and 1/2, respectively, while rates of false disjunction are 2/6 and 0, respectively. Because the probabilities of the samples are 0.3 and 0.7, the average rate of false conjunction is (0.3)(3/4)+(0.7)(1/2) = 0.575, and the average rate of false disjunction is (0.3)(2/6)+(0.7)(0) =  0.1. The average rates provide a useful characterization of a clustering algorithm under a given sampling scenario.
(0.40 MB PDF)

Figure S2 PD-based clustering is accurate relative to PID-based clustering. This graph illustrates the change in the True Conjunction Rate (solid line) and the True Disjunction Rate (dashed line) of full-length sequences clustered using PhylOTU and PD relative to the clustering obtained when the same sequences are clustered via percent identity (shown here is the PID cutoff of 0.03). Average-neighbor hierarchical clustering, the default PhylOTU setting, was used to generate these results.
(0.09 MB PDF)

Figure S3 Distribution of corresponding PD and PID pairwise distances. This figure illustrates the relationship between PD and PID distances (less than 0.2) calculated for all pairs of the 508 references sequences used in our study. The red line indicates identical distances estimated by PD and PID methods. The blue and green lines identify the clustering threshold distance of 0.03 for PD and PID, respectively. The mass of points above the red line for PD distances less than 0.03 indicates that among those pairs that are closely related as per the PD calculation, the PID method tends to estimate a larger corresponding distance (notably the points above the green line and to the left of the blue line). This observation could account for the difference in the estimated richness between the two methods. Conversely, when estimated PD distances are larger (e.g., distances greater than 0.1), the corresponding PID distance tends to be smaller. These observations are likely the result of how distances are calculated in the two approaches: PD leverages a weighted substitution model that down-weights similar substitutions and corrects for multiple substitutions, while the PID method weights all substitutions equally.
(0.26 MB PDF)

Figure S4 PhylOTU clustering of full-length sources and simulated reads is positively correlated. For each of the 25 RDP reference library-based simulations, we compared the source and simulation PD distance matrices produced by PhylOTU using a Mantel test. All 25 tests reveal a significant (p<0.05) and positive correlation coefficient. The above histogram reveals the distribution of the correlation coefficients identified through this analysis.
(0.09 MB PDF)

Figure S5 Derivation of methodological accuracy. Accuracy plots, which capture the change in the true conjunction and true disjunction rate as the simulated shotgun read clustering threshold increases relative to a fixed full-length sequence clustering threshold, were generated for several full-length sequence thresholds. We show here the accuracy plot of the source threshold of 0.03 as an example. The median true clustering and true cutting rates are represented by the solid and dashed black lines, respectively. The red line indicates the minimum tolerated accuracy, which we designate to be 80%. The most accurate read threshold is indicated by the solid blue line, which represents the point where the true clustering rate is controlled at the minimum accuracy and the true cutting rate is maximized. At least three interpretations of an optimal thresholds could be identified from this analysis, contingent upon the application: 1) the threshold where the true conjunction rate (TCR) is fixed at a controlled minimum accuracy and the true disjunction rate (TDR) is maximized, 2) the threshold where the TCR and TDR intersect, and 3) the threshold where the TDR is controlled and the TCR is maximized. The standard hypothesis testing approach is to control the type I error, which corresponds to controlling the TCR in this analysis (Methods) and results in a larger estimate of taxonomic richness.
(0.11 MB PDF)

Figure S6 Relationship between full-length sequence clustering threshold and adjusted shotgun read clustering threshold. The relationship between full-length clustering thresholds and the corresponding shotgun read clustering threshold that maximizes the True Disjunction Rate while controlling the True Conjunction Rate is plotted in black. Specifically, this curve represents a loess smoothing of the most accurate thresholds we identified from the reference library-based simulation analyses across a series of full-length sequence clustering thresholds using the procedure described in Figure S5.
(0.08 MB PDF)

Figure S7 Change in accuracy as the full-length sequence clustering threshold increases. The accuracy of clustering reads via an adjusted threshold (black line) remains high, even at relatively large full-length sequence clustering thresholds. The solid red line represents the minimum tolerated True Conjunction Rate (TCR) of 80%.
(0.07 MB PDF)

Figure S8 Accuracy with which PhylOTU clusters shotgun sequences simulated from the SILVA database relative to a full-length cutoff of 0.03. We used the SILVA-based simulation data to construct accuracy plots as described in Figure S5. We show here the accuracy plot corresponding to a full-length clustering cutoff of 0.03 as an example. The median true clustering and true cutting rates are represented by the solid and dashed black lines, respectively. The red line indicates the minimum tolerated accuracy, which we designate to be 80%. The most accurate read threshold is indicated by the solid blue line, which represents the point where the true clustering rate is controlled at the minimum accuracy and the true cutting rate is maximized. Despite the increased rigor of these simulations, PhylOTU maintains relatively high accuracy levels, albeit slightly lower than observed during the reference library-based simulation.
(0.11 MB PDF)

Figure S9 The clustering threshold affects the rate of discovery of unique and non-unique OTUs per sequence. We randomly sampled 1000 sequences from the PCR (blue lines) and shotgun (red lines) sequence libraries and counted the total number of distinct OTUs (solid lines) as well as the number of OTUs unique to each sequence library (dashed lines) identified by the sample across clustering thresholds (100 bootstraps). In regards to the total number of OTUs identified by each library, this analysis reveals that the number of OTUs discovered per sequence depreciates at similar rates in both libraries as the threshold increases. Conversely, we find that the rate of change of unique OTU discovery is not consistent between libraries: more unique OTUs per sequence are discovered in the PCR-generated sequence library at thresholds below 0.05, while the inverse is true at thresholds greater than or equal to 0.05. Notably, the number of unique OTUs per PCR sequence declines as the threshold increases at a rate similar to the total number of OTUs discovered per PCR sequence. This is not the case with shotgun data, where the slope of the unique OTUs discovered per read is much flatter. This may be the result of the increased phylogenetic diversity discovered in the shotgun library and suggests that PCR sequences tend to contribute less unique phylogenetic branch length than shotgun reads.
(0.09 MB PDF)

Figure S10 Partial alignment of shotgun sequence from uniquely metagenomic OTUs that overlap a universal SSU-rRNA primer site. Of those sequences that cluster into OTUs that are uniquely identified via analysis of shotgun sequence data (clustering threshold of 0.15), 18 overlap a universal SSU-rRNA primer site in the alignment. Here, we show the result of aligning those 18 sequences as well as the 8F and 27F primers to the INFERNAL SSU-rRNA model used in PhylOTU. We find that two sequences contain a shared C->T substitution that differentiates them from all other sequences in the alignment (red column) directly adjacent to the degenerate site in the 27F primer sequence (blue column). Incorporation of a degenerate base at this position in the universal primer sequence may enable more rigorous characterization of those lineages that harbor this C->T transition.
(0.02 MB PDF)

Table S1 Hierarchical clustering algorithms affect both clustering accuracy and richness estimates. Full-length SSU-rRNA sequences were clustered via both PID and PD approaches using three difference linkage definitions in the hierarchical clustering algorithm: nearest-neighbor (nn), average (avg), and furthest-neighbor (fn). Here, we show the results of comparing each the PD clusters (threshold of 0.03) to each of the PID clusters (threshold of 0.03) using the True Conjunction Rate (TCR) and True Disjunction Rate (TDR) calculations described in the Methods. In addition, we calculated the Richness Ratio for each comparison, which is the number of OTUs identified by the PD clustering divided by the number of OTUs identified by the PID clustering.
(0.02 MB DOC)

Table S2 Both PID and PD clustering accurately recapitulates taxonomy-guided clusters. Full-length reference sequences were clustered based on their GenBank taxonomy at the species level. These same sequences were then clustered using both the PID and PD methods by employing a distance threshold of 0.03 across three hierarchical clustering algorithms: nearest-neighbor (nn), average-linkage (avg), furthest-neighbor (fn). Each of the PID and PD clusters were compared to the taxonomy guided clusters using the True Conjunction Rate (TCR) and True Disjunction Rate (TDR) calculations described in the Methods. We also calculated the Richness Ratio, which is the number of OTUs identified by the PD or PID clustering method in question divided by the number of OTUs identified via the taxonomy-guided clusters.
(0.02 MB DOC)

Table S3 Accuracy of adjusted shotgun read clustering cutoff relative to full-length clusters when controlling the true conjunction rate and maximizing the true disjunction rate (TDR). Data was obtained from the RDP reference library-based simulations.
(0.02 MB DOC)

Table S4 Accuracy of adjusted shotgun read clustering cutoff relative to full-length clusters when controlling the true conjunction rate and maximizing the true disjunction rate (TDR). Data was obtained from the SILVA-based simulations.
(0.02 MB DOC)

Table S5 Taxonomic distribution of OTUs identified via comparison of GOS PCR-generated and shotgun-generated SSU-rRNA sequences. This table documents the frequencies at which major Bacterial taxonomic clades (approximately Bacterial phyla) were represented by sequences clustered into three different sets of OTUs identified by PhylOTU: those unique to PCR-generated SSU-rRNA (PCR), those unique to shotgun sequenced SSU-rRNA (WGS), and those reveal by both sequence libraries. The RDP taxonomy classifier was used to determine the classification of each sequence in question. Major Bacterial clades were excluded from this table if their frequency was not greater than or equal to 0.1% in at least one of the three sets of OTUs.
(0.02 MB DOC)

Text S1 Alignment quality control filters.
(0.01 MB DOC)

Text S2 MetaSim run time setting used during the PhylOTU simulation analysis.
(0.01 MB DOC)
