# Supplementary material for: PhylOTU: A High-Throughput Procedure Quantifies Microbial Community Diversity and Resolves Novel Taxa from Metagenomic Data
Source: PLoS Comput Biol. 2011 Jan 20;7(1):e1001061. doi: 10.1371/journal.pcbi.1001061 (PMC3024254; doi:10.1371/journal.pcbi.1001061)
Supplement: Figure S1 — Example showing how false conjunction and disjunction rates are calculated. In the example, two samples of reads are possible, one consisting of reads A, B, C, D, and E, and another consisting of reads C, D, F, G, and H. In reality, the set of possible samples and reads would be much larger, but for simplicity, we have chosen this smaller set. For the purposes of this example, we suppose that the probabilities of observing samples I and II are 0.3 and 0.7, respectively. At the top of each panel in grey, the partitions into OTUs based on the full length sequences are shown, while the partitions based on the simulated reads are shown in blue. The conjoined and disjoined columns give the pairs of reads placed in the same OTU and different OTUs, respectively, for the partition based on the full-length sequences. The pairs highlighted in blue are correctly conjoined or disjoined in the sample partitions. The rates of false conjunction for Samples I and II are 3/4 and 1/2, respectively, while rates of false disjunction are 2/6 and 0, respectively. Because the probabilities of the samples are 0.3 and 0.7, the average rate of false conjunction is (0.3)(3/4)+(0.7)(1/2) = 0.575, and the average rate of false disjunction is (0.3)(2/6)+(0.7)(0) = 0.1. The average rates provide a useful characterization of a clustering algorithm under a given sampling scenario. (0.40 MB PDF) [file pcbi.1001061.s001.pdf]

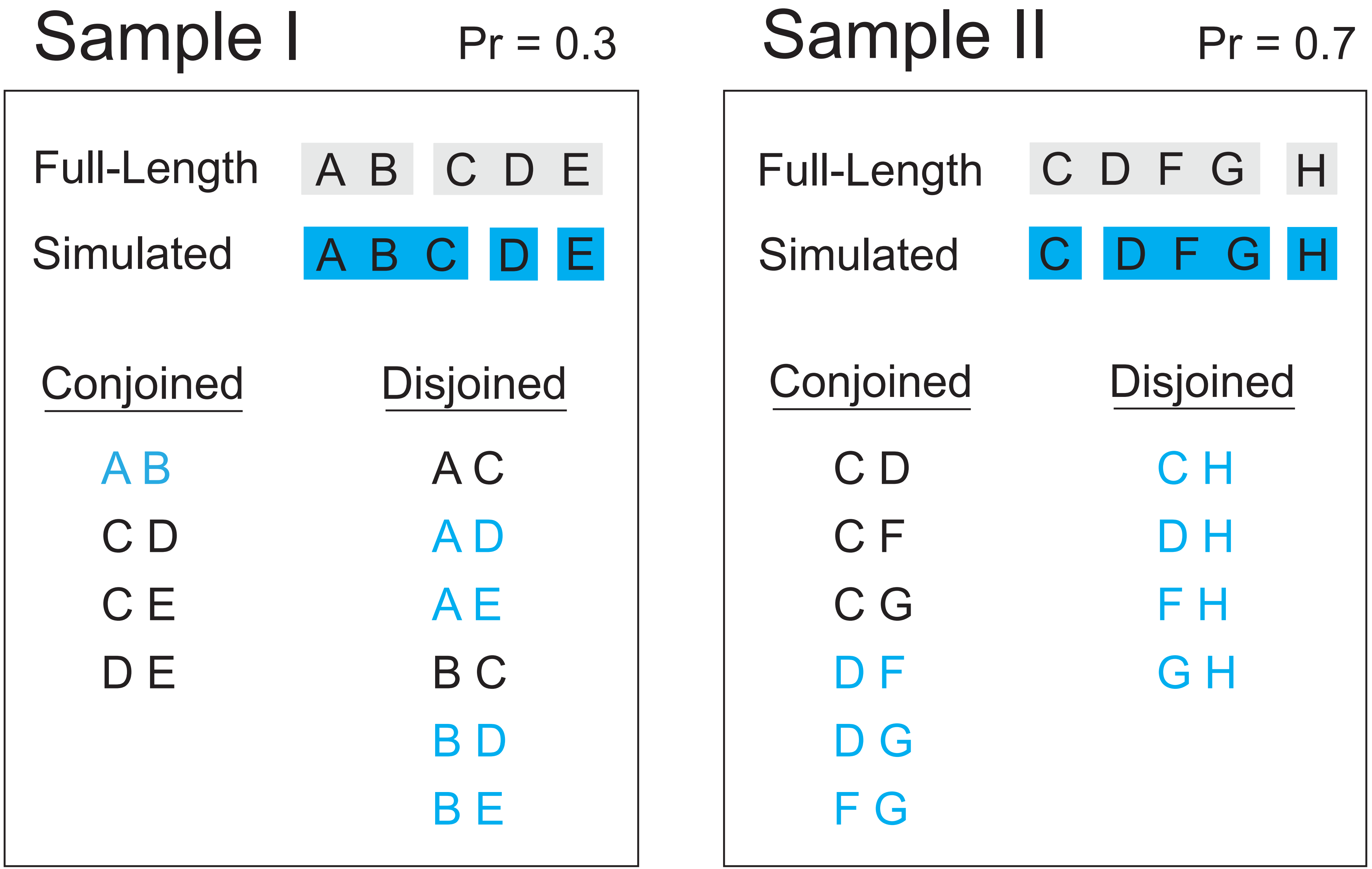

Sample I
Pr = 0.3
Full-Length
A B
C D E
Simulated
A B C
D
E
Conjoined
Disjoined
A B
C D
C E
D E
A C
A D
A E
B C
B D
B E
Sample II
Pr = 0.7
Full-Length
C D F G
H
Simulated
C
D F G
H
Conjoined
Disjoined
C D
C F
C G
D F
D G
F G
C H
D H
F H
G H
