# Supplementary figures and images for: PhylOTU: A High-Throughput Procedure Quantifies Microbial Community Diversity and Resolves Novel Taxa from Metagenomic Data
Source: PLoS Comput Biol. 2011 Jan 20;7(1):e1001061. doi: 10.1371/journal.pcbi.1001061 (PMC3024254; doi:10.1371/journal.pcbi.1001061)

**PD Clustering Accuracy Relative to PID Clusters (threshold = 0.03)**

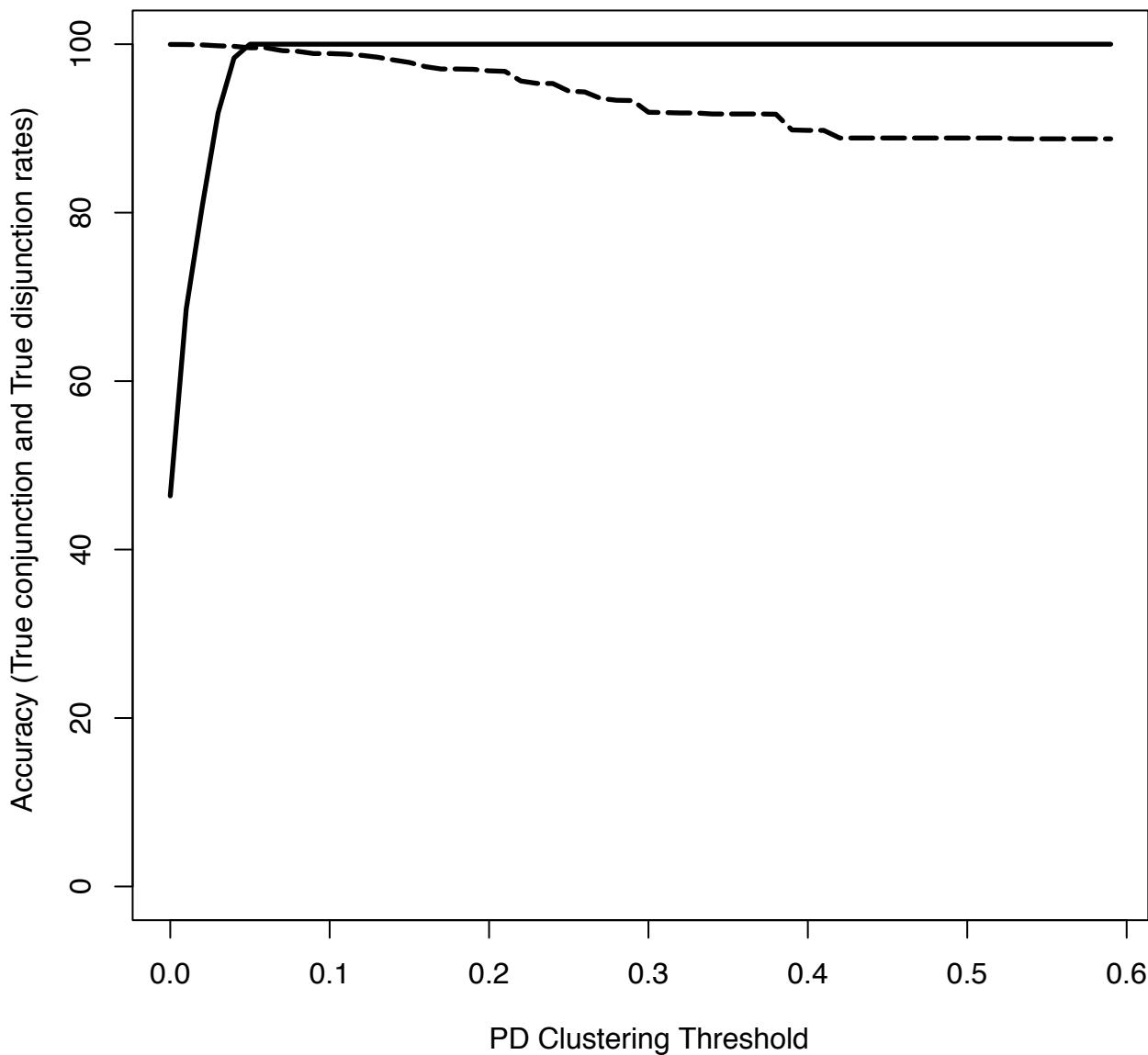

Supplement: Figure S2 — PD-based clustering is accurate relative to PID-based clustering. This graph illustrates the change in the True Conjunction Rate (solid line) and the True Disjunction Rate (dashed line) of full-length sequences clustered using PhylOTU and PD relative to the clustering obtained when the same sequences are clustered via percent identity (shown here is the PID cutoff of 0.03). Average-neighbor hierarchical clustering, the default PhylOTU setting, was used to generate these results. (0.09 MB PDF) [file pcbi.1001061.s002.pdf]

Distribution of PD and PID distances ( $< 0.2$ )

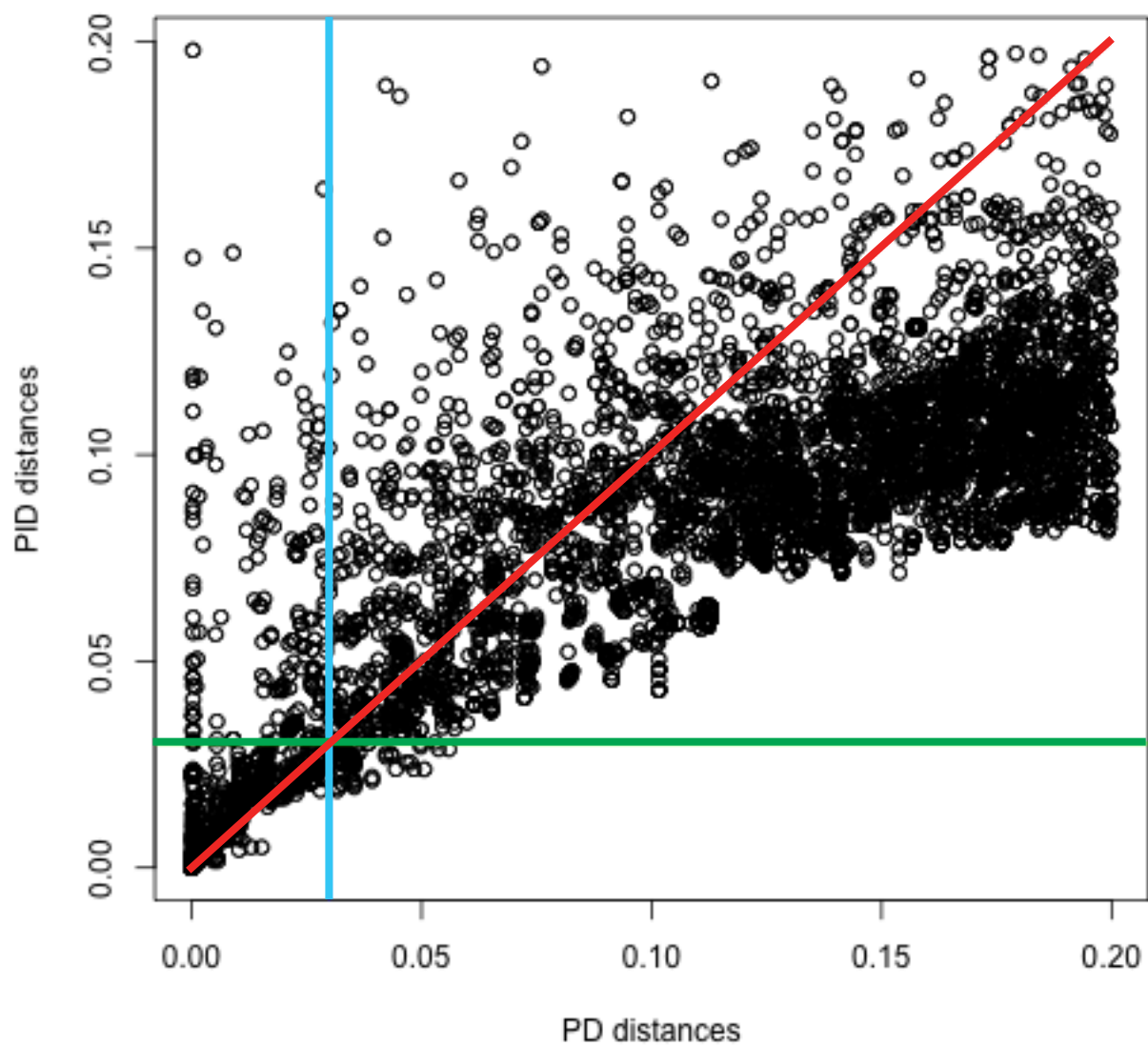

Supplement: Figure S3 — Distribution of corresponding PD and PID pairwise distances. This figure illustrates the relationship between PD and PID distances (less than 0.2) calculated for all pairs of the 508 references sequences used in our study. The red line indicates identical distances estimated by PD and PID methods. The blue and green lines identify the clustering threshold distance of 0.03 for PD and PID, respectively. The mass of points above the red line for PD distances less than 0.03 indicates that among those pairs that are closely related as per the PD calculation, the PID method tends to estimate a larger corresponding distance (notably the points above the green line and to the left of the blue line). This observation could account for the difference in the estimated richness between the two methods. Conversely, when estimated PD distances are larger (e.g., distances greater than 0.1), the corresponding PID distance tends to be smaller. These observations are likely the result of how distances are calculated in the two approaches: PD leverages a weighted substitution model that down-weights similar substitutions and corrects for multiple substitutions, while the PID method weights all substitutions equally. (0.26 MB PDF) [file pcbi.1001061.s003.pdf]

# Distribution of Mantel Test Correlation Coefficients

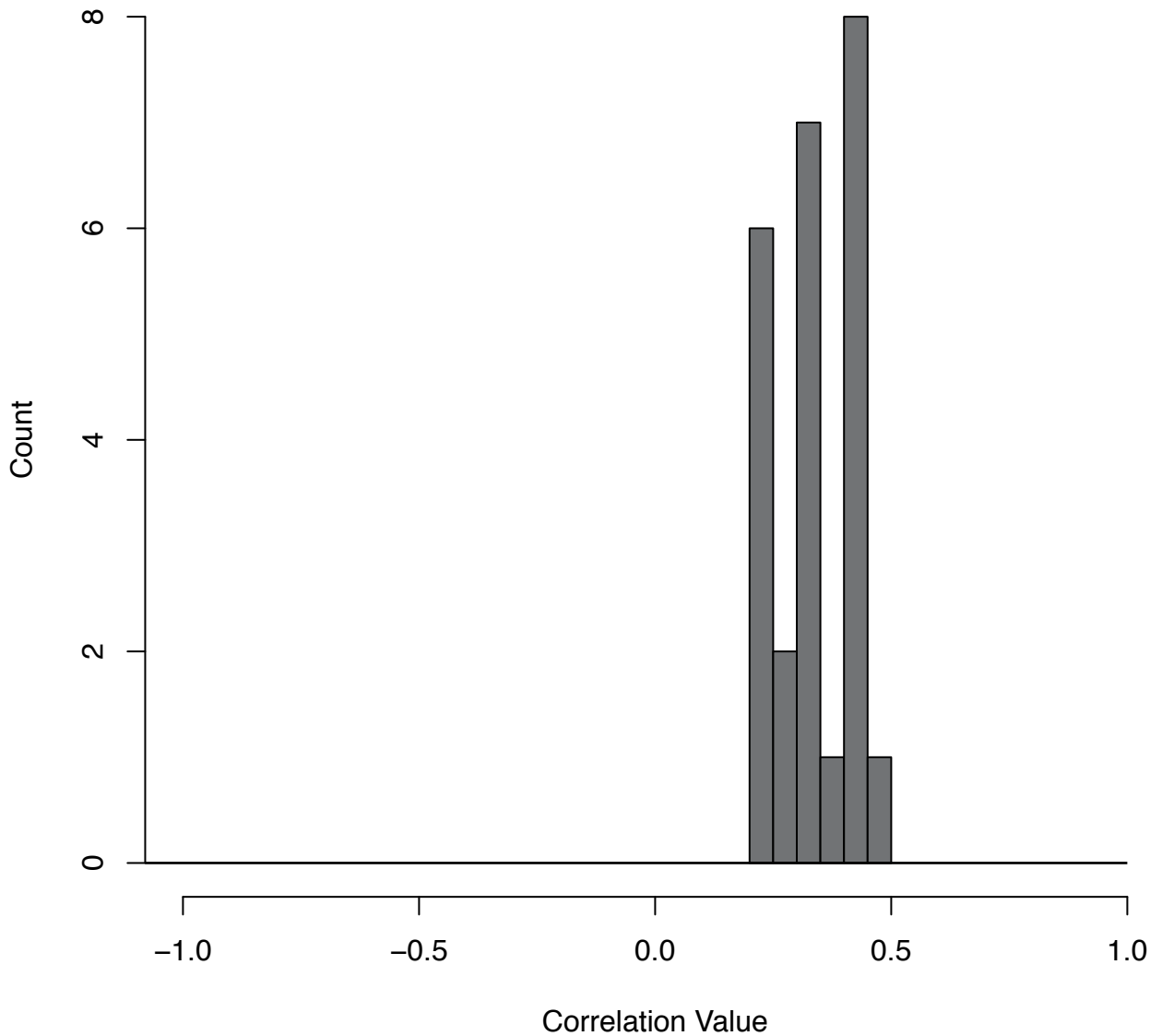

Supplement: Figure S4 — PhylOTU clustering of full-length sources and simulated reads is positively correlated. For each of the 25 RDP reference library-based simulations, we compared the source and simulation PD distance matrices produced by PhylOTU using a Mantel test. All 25 tests reveal a significant (p<0.05) and positive correlation coefficient. The above histogram reveals the distribution of the correlation coefficients identified through this analysis. (0.09 MB PDF) [file pcbi.1001061.s004.pdf]

## Accuracy for full-length phylogenetic clustering cutoff of 0.03

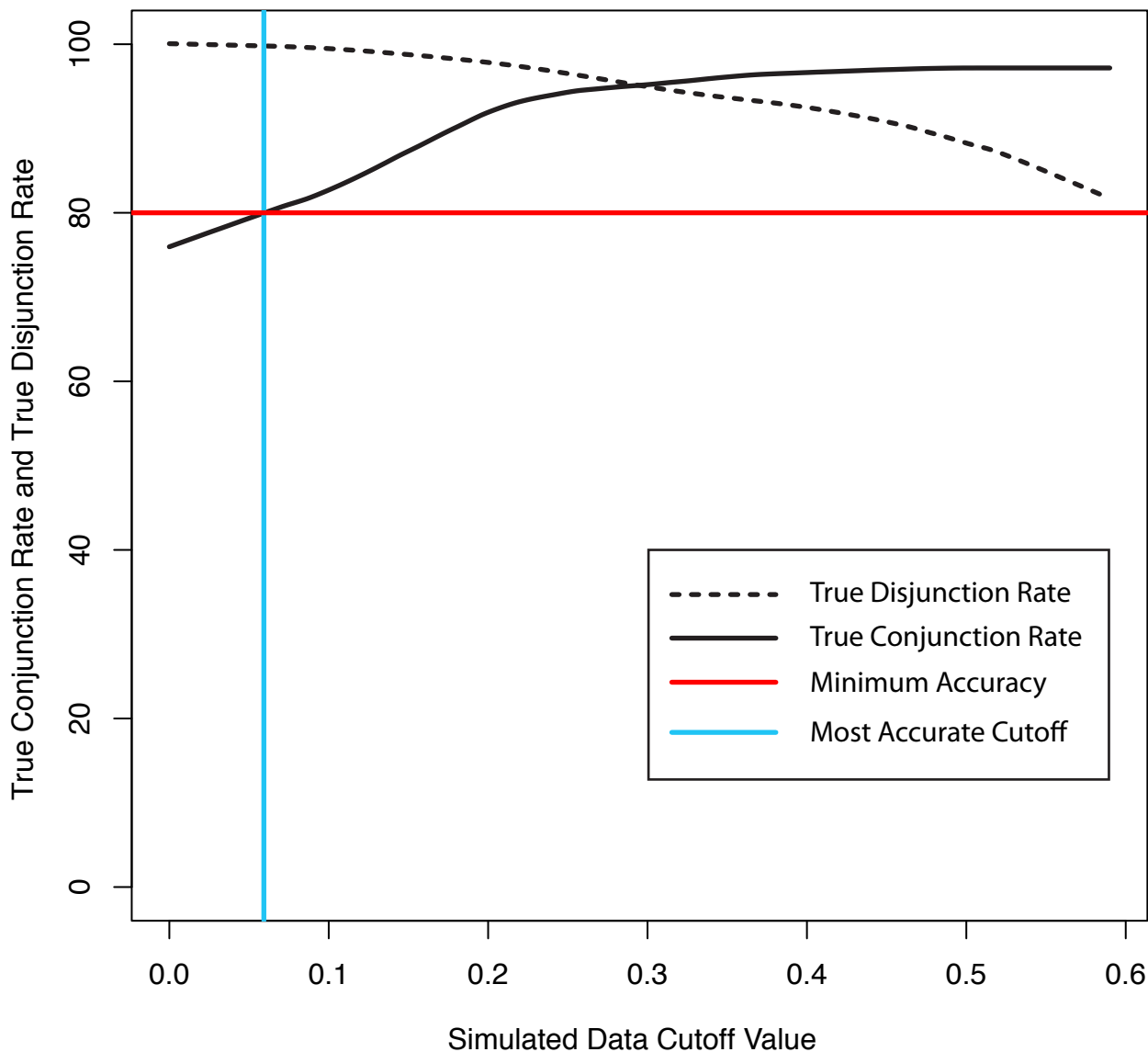

Supplement: Figure S5 — Derivation of methodological accuracy. Accuracy plots, which capture the change in the true conjunction and true disjunction rate as the simulated shotgun read clustering threshold increases relative to a fixed full-length sequence clustering threshold, were generated for several full-length sequence thresholds. We show here the accuracy plot of the source threshold of 0.03 as an example. The median true clustering and true cutting rates are represented by the solid and dashed black lines, respectively. The red line indicates the minimum tolerated accuracy, which we designate to be 80%. The most accurate read threshold is indicated by the solid blue line, which represents the point where the true clustering rate is controlled at the minimum accuracy and the true cutting rate is maximized. At least three interpretations of an optimal thresholds could be identified from this analysis, contingent upon the application: 1) the threshold where the true conjunction rate (TCR) is fixed at a controlled minimum accuracy and the true disjunction rate (TDR) is maximized, 2) the threshold where the TCR and TDR intersect, and 3) the threshold where the TDR is controlled and the TCR is maximized. The standard hypothesis testing approach is to control the type I error, which corresponds to controlling the TCR in this analysis (Methods) and results in a larger estimate of taxonomic richness. (0.11 MB PDF) [file pcbi.1001061.s005.pdf]

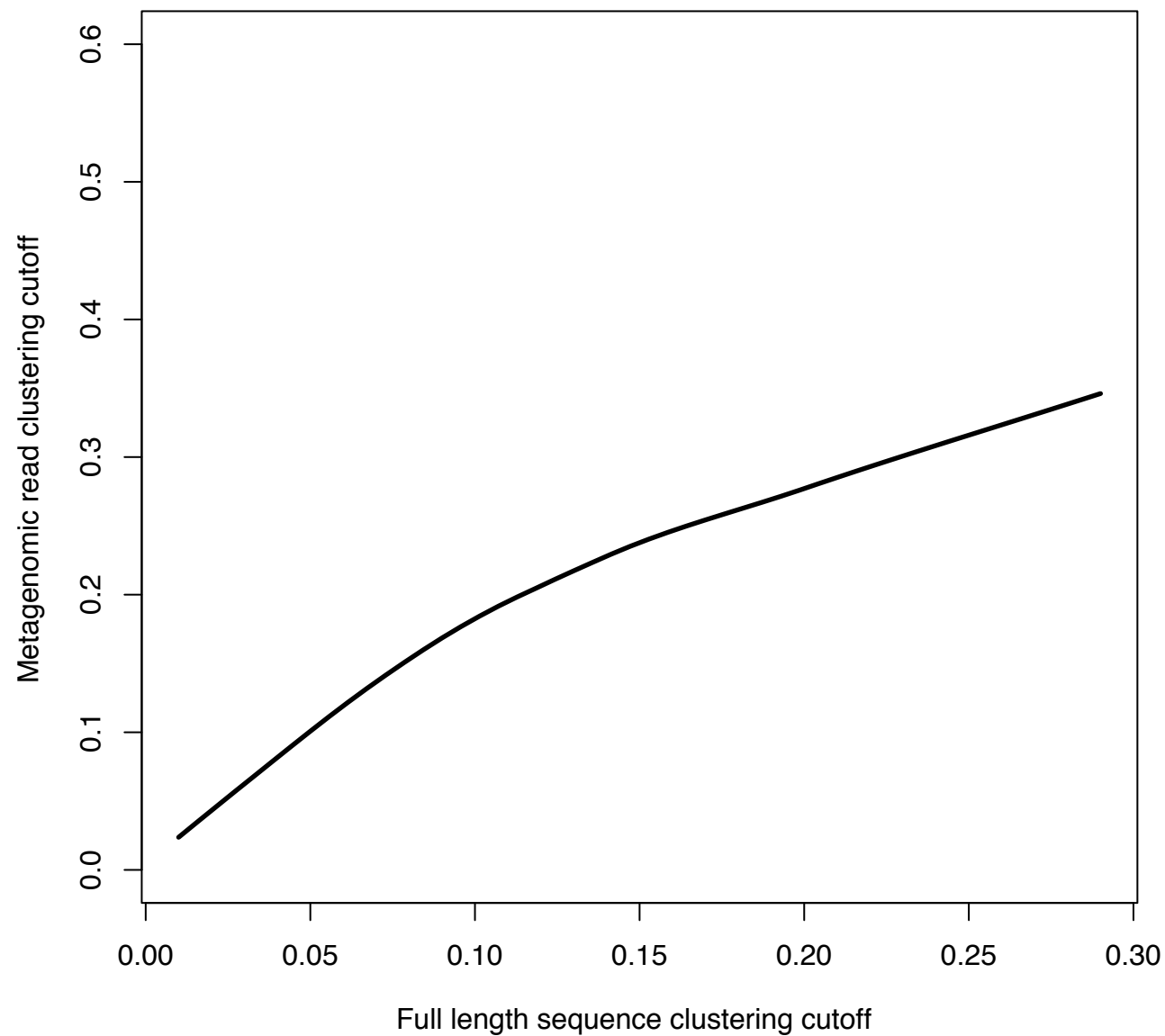

Supplement: Figure S6 — Relationship between full-length sequence clustering threshold and adjusted shotgun read clustering threshold. The relationship between full-length clustering thresholds and the corresponding shotgun read clustering threshold that maximizes the True Disjunction Rate while controlling the True Conjunction Rate is plotted in black. Specifically, this curve represents a loess smoothing of the most accurate thresholds we identified from the reference library-based simulation analyses across a series of full-length sequence clustering thresholds using the procedure described in Figure S5. (0.08 MB PDF) [file pcbi.1001061.s006.pdf]

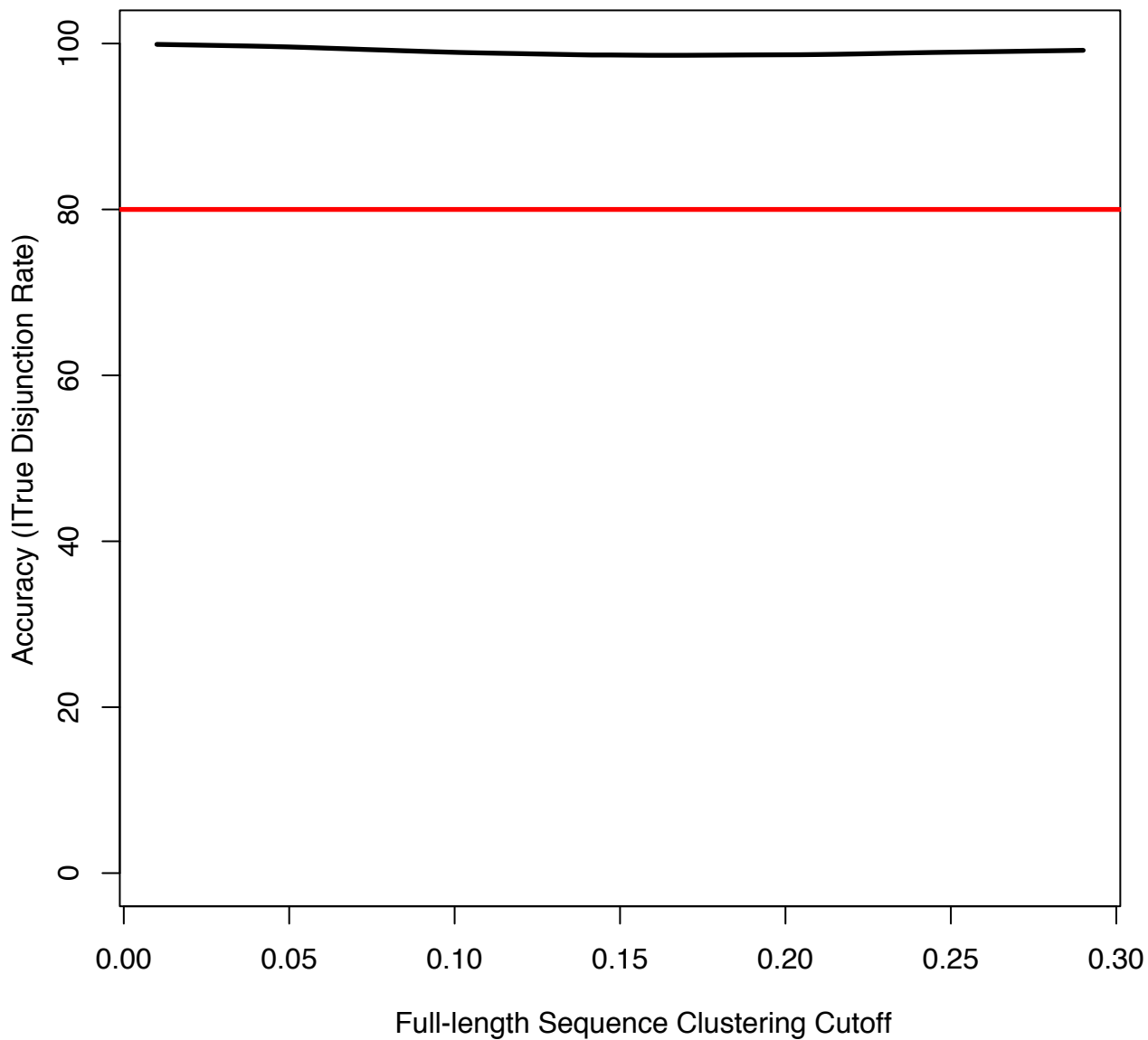

Supplement: Figure S7 — Change in accuracy as the full-length sequence clustering threshold increases. The accuracy of clustering reads via an adjusted threshold (black line) remains high, even at relatively large full-length sequence clustering thresholds. The solid red line represents the minimum tolerated True Conjunction Rate (TCR) of 80%. (0.07 MB PDF) [file pcbi.1001061.s007.pdf]

## Accuracy for full-length phylogenetic clustering cutoff of 0.03

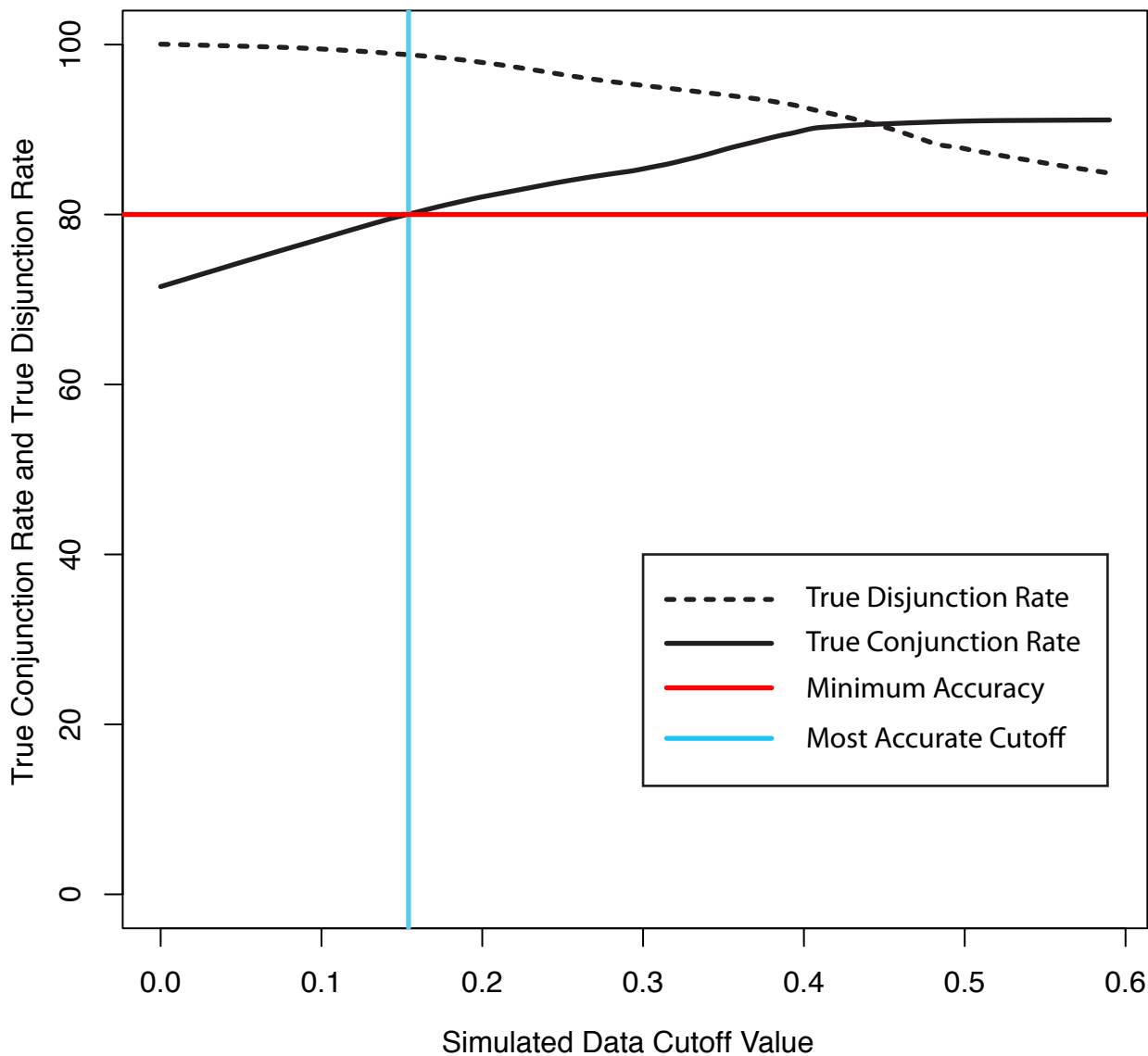

Supplement: Figure S8 — Accuracy with which PhylOTU clusters shotgun sequences simulated from the SILVA database relative to a full-length cutoff of 0.03. We used the SILVA-based simulation data to construct accuracy plots as described in Figure S5. We show here the accuracy plot corresponding to a full-length clustering cutoff of 0.03 as an example. The median true clustering and true cutting rates are represented by the solid and dashed black lines, respectively. The red line indicates the minimum tolerated accuracy, which we designate to be 80%. The most accurate read threshold is indicated by the solid blue line, which represents the point where the true clustering rate is controlled at the minimum accuracy and the true cutting rate is maximized. Despite the increased rigor of these simulations, PhylOTU maintains relatively high accuracy levels, albeit slightly lower than observed during the reference library-based simulation. (0.11 MB PDF) [file pcbi.1001061.s008.pdf]

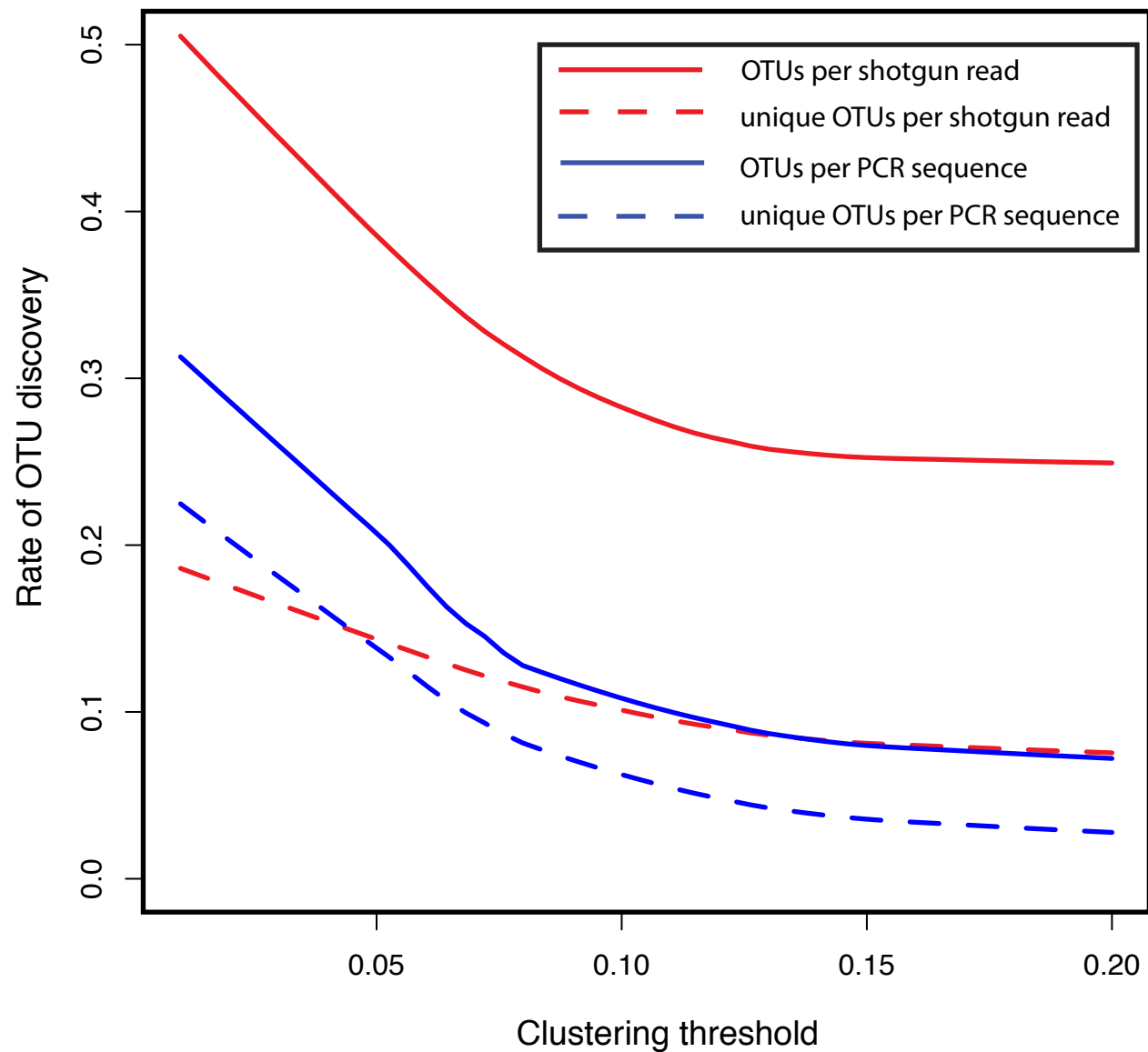

Supplement: Figure S9 — The clustering threshold affects the rate of discovery of unique and non-unique OTUs per sequence. We randomly sampled 1000 sequences from the PCR (blue lines) and shotgun (red lines) sequence libraries and counted the total number of distinct OTUs (solid lines) as well as the number of OTUs unique to each sequence library (dashed lines) identified by the sample across clustering thresholds (100 bootstraps). In regards to the total number of OTUs identified by each library, this analysis reveals that the number of OTUs discovered per sequence depreciates at similar rates in both libraries as the threshold increases. Conversely, we find that the rate of change of unique OTU discovery is not consistent between libraries: more unique OTUs per sequence are discovered in the PCR-generated sequence library at thresholds below 0.05, while the inverse is true at thresholds greater than or equal to 0.05. Notably, the number of unique OTUs per PCR sequence declines as the threshold increases at a rate similar to the total number of OTUs discovered per PCR sequence. This is not the case with shotgun data, where the slope of the unique OTUs discovered per read is much flatter. This may be the result of the increased phylogenetic diversity discovered in the shotgun library and suggests that PCR sequences tend to contribute less unique phylogenetic branch length than shotgun reads. (0.09 MB PDF) [file pcbi.1001061.s009.pdf]
