# Supplementary material for: PhylOTU: A High-Throughput Procedure Quantifies Microbial Community Diversity and Resolves Novel Taxa from Metagenomic Data
Source: PLoS Comput Biol. 2011 Jan 20;7(1):e1001061. doi: 10.1371/journal.pcbi.1001061 (PMC3024254; doi:10.1371/journal.pcbi.1001061)
Supplement: Figure S10 — Partial alignment of shotgun sequence from uniquely metagenomic OTUs that overlap a universal SSU-rRNA primer site. Of those sequences that cluster into OTUs that are uniquely identified via analysis of shotgun sequence data (clustering threshold of 0.15), 18 overlap a universal SSU-rRNA primer site in the alignment. Here, we show the result of aligning those 18 sequences as well as the 8F and 27F primers to the INFERNAL SSU-rRNA model used in PhylOTU. We find that two sequences contain a shared C->T substitution that differentiates them from all other sequences in the alignment (red column) directly adjacent to the degenerate site in the 27F primer sequence (blue column). Incorporation of a degenerate base at this position in the universal primer sequence may enable more rigorous characterization of those lineages that harbor this C->T transition. (0.02 MB PDF) [file pcbi.1001061.s010.pdf]

```
# STOCKHOLM 1.0
#=GF AU Infernal 1.0.2

JCVI_READ_1092215016641 ---------AGTTTGATTATGGCTCAGAACGAACGCTGGCGGCATGCCTC
JCVI_READ_1092351640238 -------AGAGTTTGATTCTGGCTCAGGACGAACGCTGGCGGCGTGCTTA
JCVI_READ_1095335017447 --------GAGTTTGATCATGGCTCAGAACGTACGCTGGCGGCACGCCTA
JCVI_READ_1095328034289 ------GAGAGTTTGATCCTGGCTCACGACGAACGCTGGCGGCGTGCTTT
JCVI_READ_1091141016470 -------AGAGTTTGATCCTGGCTCAGGATGAACGCTAGCGGCAGGCTTA
JCVI_READ_1091141014166 -------AGAGTTTGATCCTGGCTCAGGATGAACGCTAGCGGCAGGCTTA
8F_Turner99             -------AGAGTTTGATCCTGGCTCAG-----------------------
27F_Lane91              -------AGAGTTTGATCMTGGCTCAG-----------------------
#=GC SS_cons            ::::::::<<<<<_______>>>>>,{{{{-{{{{{{,{{{{{{{{{---
#=GC RF                 tAAatGGAGAGTTTGATCCTGGCTCAGaATGAACGCTGGCGGCgtGCCTA
```
