# Supplementary material for: PhylOTU: A High-Throughput Procedure Quantifies Microbial Community Diversity and Resolves Novel Taxa from Metagenomic Data
Source: PLoS Comput Biol. 2011 Jan 20;7(1):e1001061. doi: 10.1371/journal.pcbi.1001061 (PMC3024254; doi:10.1371/journal.pcbi.1001061)
Supplement: Table S1 — Hierarchical clustering algorithms affect both clustering accuracy and richness estimates. Full-length SSU-rRNA sequences were clustered via both PID and PD approaches using three difference linkage definitions in the hierarchical clustering algorithm: nearest-neighbor (nn), average (avg), and furthest-neighbor (fn). Here, we show the results of comparing each the PD clusters (threshold of 0.03) to each of the PID clusters (threshold of 0.03) using the True Conjunction Rate (TCR) and True Disjunction Rate (TDR) calculations described in the Methods. In addition, we calculated the Richness Ratio for each comparison, which is the number of OTUs identified by the PD clustering divided by the number of OTUs identified by the PID clustering. (0.02 MB DOC) [file pcbi.1001061.s011.doc]

Comparison of Hierarchical Clustering Methods

**Table SI 1**: Hierarchical clustering algorithms affect both clustering accuracy and richness estimates

Full-length SSU-rRNA sequences were clustered via both PID and PD approaches using three difference linkage definitions in the hierarchical clustering algorithm: nearest-neighbor (nn), average (avg), and furthest-neighbor (fn). Here, we show the results of comparing each the PD clusters (threshold of 0.03) to each of the PID clusters (threshold of 0.03) using the True Conjunction Rate (TCR) and True Disjunction Rate (TDR) calculations described in the Methods. In addition, we calculated the Richness Ratio for each comparison, which is the number of OTUs identified by the PD clustering divided by the number of OTUs identified by the PID clustering.
