# Supplementary material for: PhylOTU: A High-Throughput Procedure Quantifies Microbial Community Diversity and Resolves Novel Taxa from Metagenomic Data
Source: PLoS Comput Biol. 2011 Jan 20;7(1):e1001061. doi: 10.1371/journal.pcbi.1001061 (PMC3024254; doi:10.1371/journal.pcbi.1001061)
Supplement: Table S2 — Both PID and PD clustering accurately recapitulates taxonomy-guided clusters. Full-length reference sequences were clustered based on their GenBank taxonomy at the species level. These same sequences were then clustered using both the PID and PD methods by employing a distance threshold of 0.03 across three hierarchical clustering algorithms: nearest-neighbor (nn), average-linkage (avg), furthest-neighbor (fn). Each of the PID and PD clusters were compared to the taxonomy guided clusters using the True Conjunction Rate (TCR) and True Disjunction Rate (TDR) calculations described in the Methods. We also calculated the Richness Ratio, which is the number of OTUs identified by the PD or PID clustering method in question divided by the number of OTUs identified via the taxonomy-guided clusters. (0.02 MB DOC) [file pcbi.1001061.s012.doc]

Accuracy of PD and PID clustering relative to taxonomy-guided clustering

**Table SI 2**: Both PID and PD clustering accurately recapitulates taxonomy-guided clusters

Full-length reference sequences were clustered based on their GenBank taxonomy at the species level. These same sequences were then clustered using both the PID and PD methods by employing a distance threshold of 0.03 across three hierarchical clustering algorithms: nearest-neighbor (nn), average-linkage (avg), furthest-neighbor (fn). Each of the PID and PD clusters were compared to the taxonomy guided clusters using the True Conjunction Rate (TCR) and True Disjunction Rate (TDR) calculations described in the Methods. We also calculated the Richness Ratio, which is the number of OTUs identified by the PD or PID clustering method in question divided by the number of OTUs identified via the taxonomy-guided clusters.
