# Supplementary material for: PhylOTU: A High-Throughput Procedure Quantifies Microbial Community Diversity and Resolves Novel Taxa from Metagenomic Data
Source: PLoS Comput Biol. 2011 Jan 20;7(1):e1001061. doi: 10.1371/journal.pcbi.1001061 (PMC3024254; doi:10.1371/journal.pcbi.1001061)
Supplement: Table S3 — Accuracy of adjusted shotgun read clustering cutoff relative to full-length clusters when controlling the true conjunction rate and maximizing the true disjunction rate (TDR). Data was obtained from the RDP reference library-based simulations. (0.02 MB DOC) [file pcbi.1001061.s013.doc]

**Table S3**: Accuracy of adjusted shotgun read clustering cutoff relative to full-length clusters when controlling the true conjunction rate and maximizing the true disjunction rate (TDR)
