# Supplementary material for: PhylOTU: A High-Throughput Procedure Quantifies Microbial Community Diversity and Resolves Novel Taxa from Metagenomic Data
Source: PLoS Comput Biol. 2011 Jan 20;7(1):e1001061. doi: 10.1371/journal.pcbi.1001061 (PMC3024254; doi:10.1371/journal.pcbi.1001061)
Supplement: Table S5 — Taxonomic distribution of OTUs identified via comparison of GOS PCR-generated and shotgun-generated SSU-rRNA sequences. This table documents the frequencies at which major Bacterial taxonomic clades (approximately Bacterial phyla) were represented by sequences clustered into three different sets of OTUs identified by PhylOTU: those unique to PCR-generated SSU-rRNA (PCR), those unique to shotgun sequenced SSU-rRNA (WGS), and those reveal by both sequence libraries. The RDP taxonomy classifier was used to determine the classification of each sequence in question. Major Bacterial clades were excluded from this table if their frequency was not greater than or equal to 0.1% in at least one of the three sets of OTUs. (0.02 MB DOC) [file pcbi.1001061.s015.doc]

Taxonomic distribution of unique PCR, unique shotgun, and share PCR and shotgun OTUs

**Table S5**: Taxonomic distribution of OTUs identified via comparison of GOS PCR-generated and shotgun-generated SSU-rRNA sequences

This table documents the frequencies at which major Bacterial taxonomic clades (approximately Bacterial phyla) were represented by sequences clustered into three different sets of OTUs identified by PhylOTU: those unique to PCR-generated SSU-rRNA (PCR), those unique to shotgun sequenced SSU-rRNA (WGS), and those reveal by both sequence libraries. The RDP taxonomy classifier was used to determine the classification of each sequence in question. Major Bacterial clades were excluded from this table if their frequency was not greater than or equal to 0.1% in at least one of the three sets of OTUs
