# Supplementary material for: PhylOTU: A High-Throughput Procedure Quantifies Microbial Community Diversity and Resolves Novel Taxa from Metagenomic Data
Source: PLoS Comput Biol. 2011 Jan 20;7(1):e1001061. doi: 10.1371/journal.pcbi.1001061 (PMC3024254; doi:10.1371/journal.pcbi.1001061)
Supplement: Text S1 — Alignment quality control filters. (0.01 MB DOC) [file pcbi.1001061.s016.doc]

Alignment Quality Control Filters

Our workflow incorporates several filters that minimize the possibility of creating spurious alignments. First, as described in the Results, sequences less than 100 bp in length are removed from the alignment. In addition, to reduce the possibility that a read has been stretched out across the entire profile length, as opposed to being accurately and locally aligned to a specific profile subregion, we remove sequences that have more than 50 gap characters bounded by residues (so called “internal-gaps”). We find that the -sub option in the cmalign (INFERNAL) is rigorous enough that this particular filter is rarely activated. Furthermore, to ensure that only SSU-rRNA sequences from the appropriate phylogenetic domain (e.g., Bacteria, Archaea, Eukaryotes), are incorporated in the alignment, we require that sequences exhibit a cmalign alignment score per residue greater than 1.0. Analysis of the RDP full-length Bacteria and Archaea reference sequences as well as RDP taxonomy classification of PhylOTU identified GOS SSU-rRNA reads indicates that this threshold is sensitive enough to discriminate between phylogenetic domains. Finally, we remove alignment columns that have more that 75% gap characters.
