# Supplementary material for: PhylOTU: A High-Throughput Procedure Quantifies Microbial Community Diversity and Resolves Novel Taxa from Metagenomic Data
Source: PLoS Comput Biol. 2011 Jan 20;7(1):e1001061. doi: 10.1371/journal.pcbi.1001061 (PMC3024254; doi:10.1371/journal.pcbi.1001061)
Supplement: Text S2 — MetaSim run time setting used during the PhylOTU simulation analysis. (0.01 MB DOC) [file pcbi.1001061.s017.doc]

MetaSim run time setting used during the PhylOTU simulation analysis

MetaSim was run with the following parameter settings: the Sanger error model (which was used for its command-line flexibility to mimic 454-type reads without error), no sequencing error, normal read-length distribution with a mean of 500bp and standard deviation of 120bp, normal clone-length distribution with a mean of 500bp and standard deviation of 120bp, and the uniform sequence weights option set to false. Due to interactions in the MetaSim parameter settings, the reads generated had an actual mean length of 434 bp, with a standard deviation of 99 bp, which approximates a 454-like read-length distribution.

For more on MetaSim, see Richter DC, Ott F, Auch AF, Schmid R, Huson DH. (2008) Metasim: a sequencing simulator for genomics and metagenomics. PLoS ONE, 3:e3373.
